# Binary image acquisition and texture parameter calculation of asphalt pavement based on a U-Net model

**DOI:** 10.1371/journal.pone.0354127

**Published:** 2026-07-22

**Authors:** Fengwei An, Haoran Jiang, Yulong Zhao, Lei Fang, Shujing Dong, Wenpeng Du, Guohua Wu, Wei Liu, Zhenglong Lv, Hao Liang

**Affiliations:** 1 China Communications Construction Jiangsu Highway Engineering Co., Ltd., Nanjing, China; 2 National Engineering Research Center for Novel Road Materials, Nanjing, China; 3 Shandong Pengcheng Road and Bridge Group Co., Ltd., Jining, China; 4 School of Transportation and Civil Engineering, Shandong Jiaotong University, Jinan, China; 5 Jining-Jizhou Expressway Co., Ltd., Jining, China; Henan Polytechnic University, CHINA

## Abstract

Current methods for detecting and evaluating pavement skid resistance vary widely, yet each has its own scope of applicability and inherent limitations. Therefore, this paper proposes a method based on U-Net model segmentation to obtain binary images of asphalt pavement surfaces, enabling precise calculation of pavement texture distribution parameters. A dataset required for model training and testing was constructed, and preprocessing was performed on forward-collected asphalt pavement images to eliminate noise interference. Based on the established three-dimensional asphalt pavement model, reverse engineering is employed to obtain binary images of the pavement surface as target images for model training. A U-Net semantic segmentation model is constructed to train the segmentation of asphalt pavement images, distinguishing between the aggregate and void components of the pavement. The feature parameters of the binary images generated by U-Net segmentation are calculated and correlated with the average texture depth measured using the sand patch method. Results indicate that the U-Net model achieved an F1-score of 0.7214 on the validation set, demonstrating satisfactory segmentation performance for subsequent texture parameter extraction. The correlation coefficient R² between the mean texture depth (MTD) calculated from the binary image and the sand patch method reached 0.85, while the correlation coefficient between the fractal dimension and MTD-1 was 0.86. Both correlations were statistically significant at the 95% confidence level. The proposed method can provide effective technical support for texture-based skid resistance evaluation of asphalt pavements.

## 1. Introduction

To enhance road traffic safety, countries worldwide invest significant resources, finances, and time in evaluating road safety performance, aiming to provide timely and reasonable assessments and monitoring of road service functions [1–3]. According to regulatory standards, asphalt pavements must provide efficient, comfortable, safe, and stable service for vehicular traffic, meeting requirements for smoothness, high-temperature stability, water resistance, crack resistance, structural strength, and skid resistance [4–7]. However, in recent years, traffic accidents on China’s asphalt pavements have become frequent. Key influencing factors include road-related elements (such as pavement structure, materials, alignment, and skid resistance), vehicle-related factors (vehicle speed, tire type), human factors (driving skill, physical condition, psychological state), and environmental conditions (temperature, wind, rain, ice, and snow). Among these, road skid resistance is the most critical consideration for road safety [8,9].

For the skid resistance and friction performance of asphalt pavements, the texture structure of the road surface is the most critical characteristic factor [10–12]. As the part directly contacting vehicle tires and providing surface friction, the roughness and friction force of pavement texture directly impact driving stability and safety. Therefore, accurately, reasonably, and comprehensively evaluating pavement texture distribution is particularly crucial for studying skid resistance [13–16].

Currently, skid resistance is typically evaluated using texture depth and friction coefficients. However, traditional testing methods are limited to fixed-point measurements and are highly susceptible to human factors [17,18]. Additionally, newly constructed asphalt pavements inherently exhibit unevenness. Traditional fixed-point measurements fail to comprehensively reflect the overall texture distribution and skid resistance of the pavement, potentially leading to misjudgments of actual road conditions and compromising road safety [19,20].

Advancements in image acquisition/processing and laser scanning technologies have significantly improved pavement texture and skid resistance assessment methods. Machine vision-based analysis of pavement texture structures to evaluate skid resistance has emerged as a recent research focus [21,22].

Research indicates that models such as artificial neural networks (ANN), random forests (RF), support vector machines (SVR), and extreme gradient boosting (XGBoost) can effectively establish nonlinear mapping relationships between multiple factors and performance metrics. These models demonstrate high predictive accuracy and strong generalization capabilities in complex engineering problems like concrete compressive strength. Some studies further enhance prediction accuracy and reveal the mechanisms of key influencing factors by integrating deep learning models with interpretability analysis methods. These findings demonstrate that data-driven intelligent models possess strong modeling capabilities and application potential in analyzing complex engineering problems [23–25].

Addressing the limitations of existing pavement inspection methods in automation and spatial information acquisition, this paper proposes a novel approach for evaluating asphalt pavement skid resistance by coupling 3D reconstruction technology with deep learning algorithms. Its core logic is as follows: First, multi-angle images are captured and processed using SFM algorithms to generate three-dimensional pavement models with true geometric features, overcoming the lack of spatial depth information in traditional two-dimensional images. Second, an innovative “reverse mapping” mechanism is proposed to extract binary images from the 3D models as ground truth labels for training the U-Net model. This addresses the subjectivity and low efficiency associated with traditional manual annotation, contributing to improved pavement texture recognition capability and supporting texture-based pavement skid resistance evaluation. Finally, the trained U-Net model enables automatic analysis of pavement aggregates and voids, allowing the calculation of key metrics such as mean texture depth (MTD) and fractal dimension. Through validation against traditional sand patch method measurements, this study aims to propose a deep learning-based pavement texture extraction method for efficient, non-contact, and relatively high-precision texture characterization and texture-based skid resistance evaluation.

## 2. Methods

**Fig 1:**
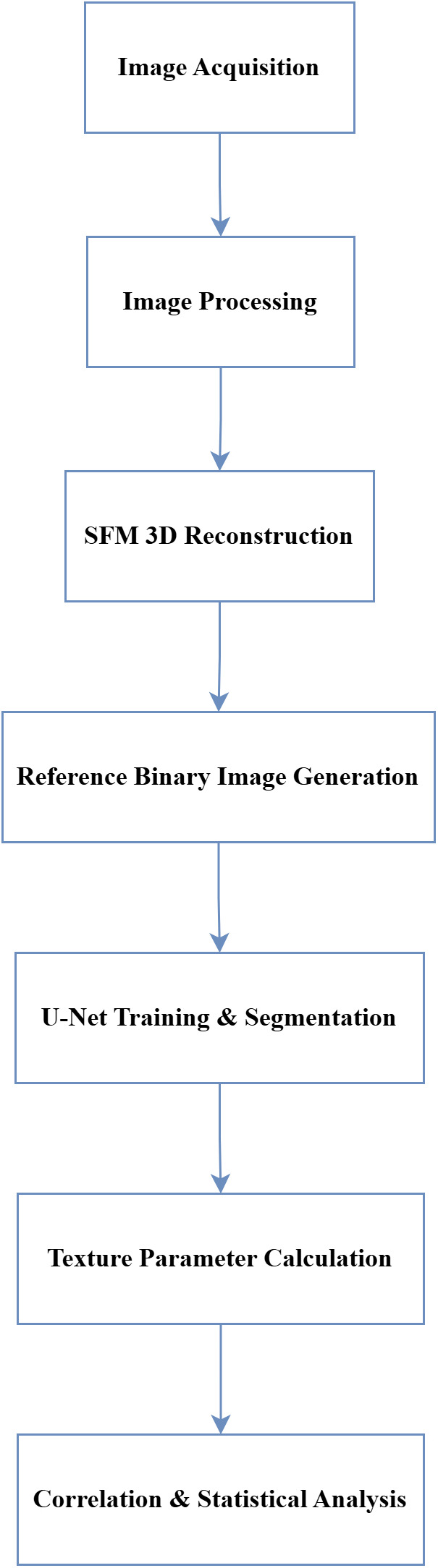
Overall workflow of the proposed method.

This study developed an integrated evaluation system for pavement skid resistance that combines 3D reconstruction with deep learning. The complete workflow is illustrated in Fig 1.

### 2.1. Deep learning-based asphalt pavement image segmentation

#### 2.1.1. U-Net network architecture.

The most distinctive feature of the U-Net model is its U-shaped architecture. The network incorporates a left-side contraction path (encoder) that extracts global image information and a right-side decompression expansion path (decoder) [26–29].

During encoding, the process involves 4 downsampling operations, 5 convolutions, and 4 max pooling operations. Each convolutional layer uses a 3 × 3 kernel size and the ReLU activation function to extract local image features. During downsampling, a 2 × 2 max pooling layer is applied to the image. This helps effectively reduce computational complexity by shrinking feature map dimensions and minimizing the risk of overfitting in the network. During convolution, effective convolution is employed to maintain a relatively stable number of feature maps while moderately reducing resolution. Each subsampling operation halves the feature map dimensions while doubling the number of channels, enhancing the model’s expressive power alongside reduced feature map size.

During decoding, four 2 × 2 deconvolutions double the feature map size while halving the number of channels. The upsampled feature maps are then fused with corresponding feature maps from the left layer via skip connections. Since feature maps on opposite sides of the same level may differ in size, the U-Net first expands smaller feature maps to match the size of larger ones before concatenation. Following fusion, subsequent convolutions, pooling, and activation functions generate the final output feature map. The network’s final layer employs a 1 × 1 convolution kernel to map each 64-dimensional feature vector to the output layer.

#### 2.1.2. Dataset construction.

(1) Image Acquisition

**Fig 2:**
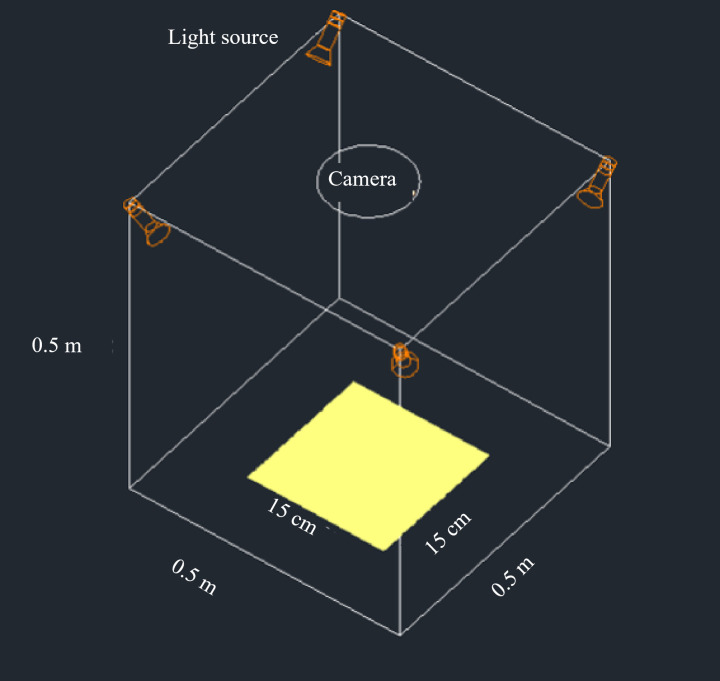
Image acquisition.

The image acquisition setup for asphalt pavement surfaces is illustrated in Fig 2. This study employs a Charge Coupled Device industrial camera to capture images of asphalt pavement surfaces. The vertical acquisition position should maintain the lens axis perpendicular to the asphalt surface as much as possible. The camera is positioned approximately 50 cm above ground level. A 15 cm × 15 cm square scale with cutouts is used as the image reference, facilitating subsequent range calibration and segmentation processing of the captured photographs.

(2) Acquisition of Three-Dimensional Asphalt Pavement Models

Based on the Structure from Motion (SFM) algorithm, three-dimensional reconstruction is performed on asphalt pavement images captured from multiple viewpoints. The specific workflow includes: first, feature point extraction and matching of multi-view images to estimate camera pose parameters and complete image registration, generating a sparse point cloud; subsequently, dense point cloud reconstruction, mesh modeling, and texture mapping are performed to obtain a three-dimensional model of the asphalt pavement surface.

This study employs Agisoft Metashape software to implement the modeling process. Feature detection utilizes a scale-invariant feature-tracking (SIFT)-based matching method, while camera parameters are obtained through joint optimization of intrinsic and extrinsic parameters. To ensure reconstruction accuracy, the results are validated through scale calibration and model flatness testing. Relevant algorithm principles and implementation details can be found in Reference [30].

(3) Inverse acquisition of a binary image

Select the top-down view of the surface layer from the 3D reconstruction model of the road surface for binarization processing, serving as the standard image for training the U-Net network model. The specific steps are as follows:

a). Extract the top-down view of the surface layer from the 3D reverse-reconstructed road texture model through Matlab programming;
b). Convert the acquired top-down view of the road texture surface layer into a binary image.

(4) Dataset Partitioning

The core purpose of dataset partitioning is to ensure an objective evaluation of model performance while reducing the risk of overfitting and data leakage [31]. In this study, a hold-out validation strategy was adopted, which is commonly used in deep learning-based pavement image analysis studies. A total of 180 asphalt pavement images were randomly divided into training and validation sets at a ratio of 8:2 [32], where 144 images were used for model training and 36 images were used for validation. All images were resized to 512 × 512 pixels before training.

Regarding the dataset scale, although the number of pavement images is relatively limited, the collected images covered different pavement texture and lighting conditions, which helps improve dataset diversity under the investigated conditions. Moreover, the extracted texture parameters maintained good agreement with the measured values obtained by the sand patch method, indicating that the current dataset is suitable for pavement texture characterization under the investigated conditions.

#### 2.1.3 Experimental environment and parameter settings.

(1) Experimental Setup

This study implements the U-Net network model based on the PyTorch framework. The dataset images were uniformly resized to 512 × 512 pixels as described earlier. Training and validation sets were allocated according to the original dataset proportion. The validation set was used to assess whether the model exhibited overfitting during training. Model parameter settings are detailed in Table 1.

**Table 1:** Training parameter settings for U-Net image segmentation of asphalt pavement.

Parameter	Value
Batch size	4
Epoch	100
Initial learning rate	0.0001

(2) Loss Function Configuration

The loss function determines the model’s training methodology. Its fundamental purpose is to quantify the discrepancy between the model’s predicted values and actual values. During model training, it guides the optimization algorithm in adjusting parameters to minimize the loss value, thereby enhancing the model’s predictive performance [33,34]. Since asphalt pavement image segmentation involves only two classes (aggregate and void), it can be treated as a binary classification problem. Therefore, the binary cross-entropy loss function is employed as the loss function during neural network training.


$$\begin{array}{c} L=-\frac{\text{1}}{N}\sum_{i=\text{1}}^{N}\left[y_{i}\;\text{log}\left(p_{i}\right)+\left(\text{1}-y_{i}\right)\text{log}\left(\text{1}-p_{i}\right)\right] \end{array}$$ (1)

In the equation, N denotes the total number of samples (or total number of pixels), y_i_ represents the true label of the i-th sample, and p_i_ denotes the probability value predicted by the model for the positive class. The negative sign ensures the loss function remains non-negative, enabling the model to optimize parameters during training by minimizing the loss function.

(3) Model Evaluation Metrics

This paper employs accuracy (A), precision (P), recall (R), F1-score and IoU (Intersection over Union) to evaluate the model [35,36,37]. The calculation equations are as follows:


$$\begin{array}{c} A=\frac{TP+TN}{TP+TN+FP+FN} \end{array}$$ (2)

$$\begin{array}{c} P=\frac{TP}{TP+FP} \end{array}$$ (3)

$$\begin{array}{c} R\text{=}\frac{TP}{TP+FN} \end{array}$$ (4)

$$\begin{array}{c} F1=\frac{2\times P\times R}{P+R} \end{array}$$ (5)

$$\begin{array}{c} IoU=\frac{TP}{TP+FP+FN} \end{array}$$ (6)

In the equation, (True Positives) represents the number of pixels where the model correctly predicts aggregate particle pixels as aggregate particle pixels in the detection results. (False Positives) represents the number of background pixels incorrectly predicted as aggregate pixels. (False Negatives) represents the number of aggregate pixels incorrectly predicted as background pixels. True Negatives (TN) represent the number of background pixels correctly predicted as background pixels.

### 2.2. Skid resistance evaluation indicators

This study employs two texture-related parameters, Mean Texture Depth (MTD) and fractal dimension, to characterize asphalt pavement surface texture associated with skid resistance. MTD represents the average depth of surface macrotexture irregularities and is commonly used to reflect pavement texture and drainage characteristics. The fractal dimension, derived from fractal theory, is used to describe the complexity and roughness of pavement surface morphology, where higher values generally indicate rougher texture features.

To evaluate the relationship between the extracted texture parameters and measured pavement texture characteristics, Pearson correlation analysis was conducted between the calculated parameters and the MTD values obtained using the sand patch method. The corresponding p-values and 95% confidence intervals were also calculated to further assess the statistical significance of the reported correlations.

#### 2.2.1. Mean texture depth.

(1) Sand Patch Method for Measuring Texture Depth

**Fig 3:**
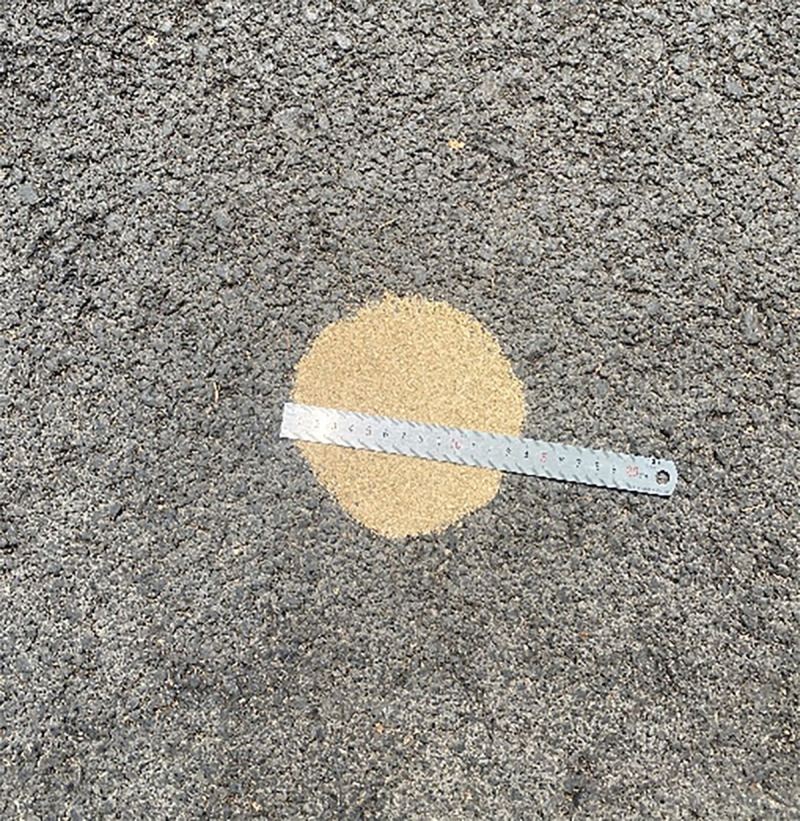
Measurement of texture depth using the sand patch method.

Chinese domestic standards such as the Highway Asphalt Pavement Design Specification (JTG D50-2017) [38] utilize the average texture depth as a surface texture characteristic parameter for evaluating the skid resistance of asphalt pavements. The basic principle of the manual sand patch method is to spread fine sand with a particle size of 0.15–0.3 mm on the pavement surface. The mean texture depth is then calculated based on the ratio of the sand volume embedded in the surface to the area covered [37], as illustrated in Fig 3.

The surface texture depth of the pavement is calculated using equation (7):


$$\begin{array}{c} TD=\frac{1000v}{\pi d^{\text{2}}/4} \end{array}$$ (7)

In the equation, TD represents the pavement surface texture depth (mm), v is the sand volume (25 cm³), and d is the average diameter of the spread sand (mm).

(2) Computing Texture Depth from Binarized Images

The binarized image segmented by the U-Net is compared with the pre-processed grayscale image. The average gray value of the white convex areas within the binarized image [39] is calculated from the corresponding gray values in the grayscale image. Simultaneously, the average gray value of the corresponding black concave areas is calculated. Therefore, the difference between the average gray values of these two parts is used to characterize the texture depth of the asphalt pavement.

By statistically counting the number of pixels in the digital image that correspond to the standard scale markings of the physical calibration strip within the plane region, the pixel equivalent length (or pixel scale) can be obtained, as shown in equation (8).


$$\begin{array}{c} \varepsilon =\frac{l}{m} \end{array}$$ (8)

In the equation, *ε* represents the pixel equivalent length, *l* is the actual calibration scale standard, and m is the corresponding number of pixels.

Through morphological processing, the number of pixels in each convex subregion can be obtained, and the actual area of each subregion can then be calculated using the equation (9).


$$\begin{array}{c} S_{i}=\varepsilon^{\text{2}}\times N_{i} \end{array}$$ (9)

In the equation, $S_{i}$ is the actual area of the i-th convex subregion, *ε* is the pixel equivalent length, and $N_{i}$ is the total number of pixels in the i-th convex subregion.

In the grayscale image, the grayscale values corresponding to the pixel positions of the white convex regions in the binary image are denoted as $G_{ij}^{\text{1}}$, with a total of k pixels. The grayscale values corresponding to the pixel positions of the black concave regions are denoted as $G_{ij}^{\text{0}}$, with a total of n pixels. The equations for calculating the average grayscale values of each region are:


$$\begin{array}{c} {\overset{―}{G}}_{\text{1}}=\frac{\sum G_{ij}^{\text{1}}}{k} \end{array}$$ (10)

$$\begin{array}{c} {\overset{―}{G}}_{\text{0}}=\frac{\sum G_{ij}^{\text{0}}}{n} \end{array}$$ (11)

The difference between the grayscale values of the two regions is then obtained as:


$$\begin{array}{c} {\overset{―}{G}}_{1-0}=\frac{\sum G_{ij}^{\text{1}}}{k}-\frac{\sum G_{ij}^{\text{0}}}{n} \end{array}$$ (12)

The texture depth value corresponding to the unit grayscale difference is given by the following equation (13):


$$\begin{array}{c} {\overset{―}{G}}_{a}=\frac{{\overset{―}{G}}_{1-0}}{MTD} \end{array}$$ (13)

#### 2.2.2 Fractal Dimension.

According to the definition of fractal dimension, the calculation process involves covering the image to be analyzed with boxes of side length r, recording the number of non-empty boxes obtained, and then progressively reducing the box size r. As r approaches 0, the box-counting fractal dimension of the image is obtained [40–42]. The specific equation is as follows:


$$\begin{array}{c} D_{Box}=\underset{r\to 0}{lim}\frac{\text{log}\left(N_{r}\right)}{\text{log}\left(1/r\right)} \end{array}$$ (14)

In the equation, $D_{Box}$ is the box-counting dimension, r is the box side length, and *N(r)* is the number of non-empty boxes. Since the limit form in the equation (14) is difficult to solve, the least squares method is used to perform linear fitting of the data points for convenience in subsequent calculation, yielding the equation (15), *α* in which $D_{Box}$ represents the box-counting dimension.


$$\begin{array}{c} ln\;N\left(r\right)=aln\left(\frac{\text{1}}{r}\right)+b \end{array}$$ (15)

## 3. Results

### 3.1. Image acquisition, preprocessing and binary images from inverse acquisition

#### 3.1.1. Image acquisition.

**Fig 4:**
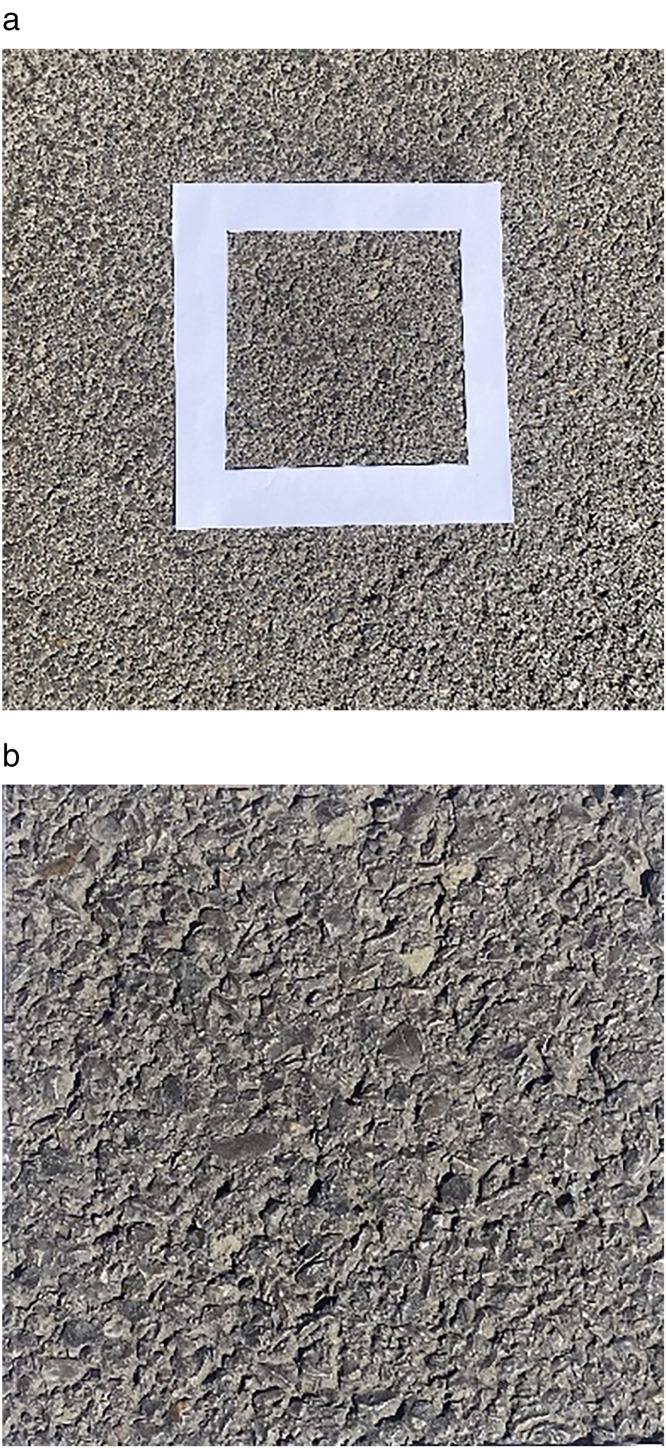
Raw asphalt pavement image captured. (a) On-site capture of raw panoramic images. (b) Raw sample images prior to preprocessing.

The raw images captured are shown in Fig 4, clearly revealing the distribution patterns and void characteristics of the aggregate on the asphalt pavement surface. This provides foundational data for subsequent segmentation and texture calculations.

#### 3.1.2. Preprocessing.

**Fig 5:**
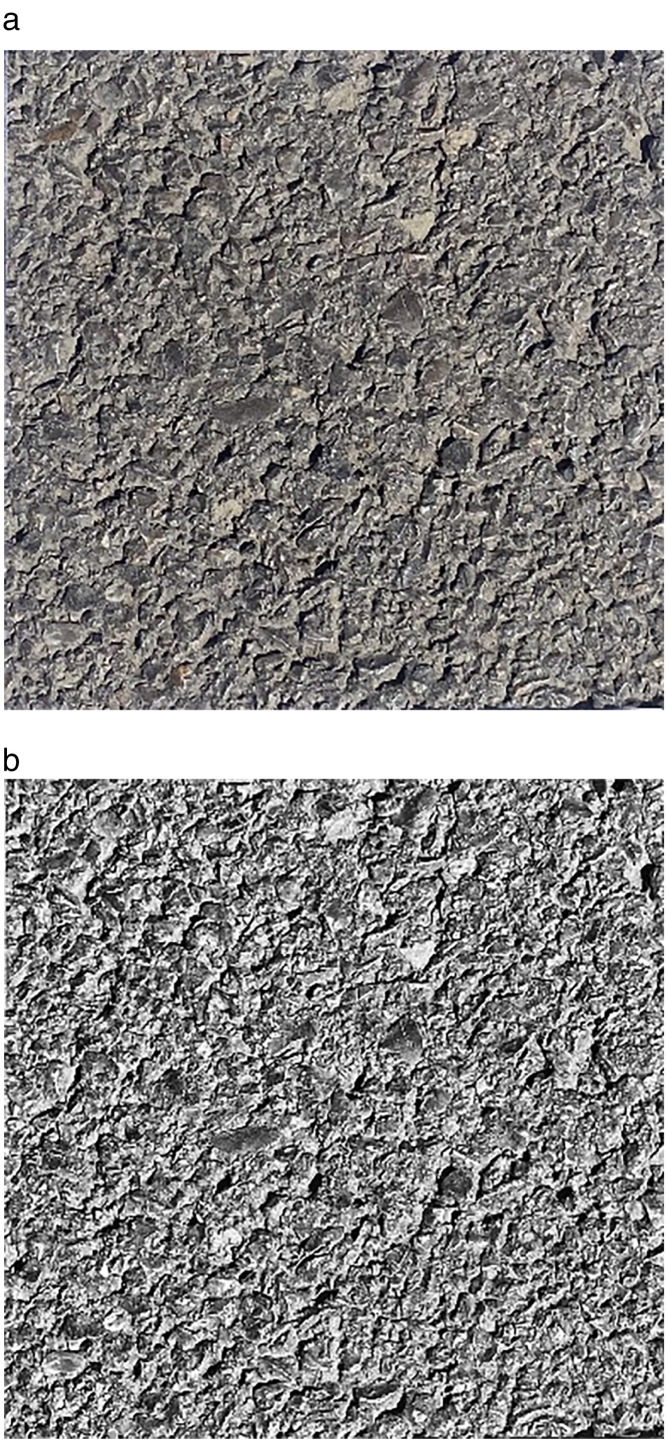
Asphalt pavement image pretreatment. (a) Raw sample images prior to preprocessing. (b) Preprocessed image samples.

To minimize interference from ambient lighting and noise on subsequent segmentation results, a series of preprocessing operations was applied to the captured images. These primarily included: grayscaling, noise filtering, and contrast enhancement. The preprocessed image comparison is shown in Fig 5. It can be observed that the edges in the preprocessed images are sharper and the brightness distribution is more uniform, providing more stable input samples for subsequent deep learning segmentation.

#### 3.1.3. Three-dimensional reconstruction.

Using multi-view image feature matching and sparse point cloud generation algorithms, photogrammetric modeling software was employed to perform 3D reverse reconstruction of the asphalt pavement’s two-dimensional texture images. Following the completion of the three-dimensional reverse reconstruction process, the processed point cloud data were utilized to reconstruct the 3D model. The primary objective was to convert the sparse point cloud data into a complete three-dimensional model, followed by its optimization and visualization.

**Fig 6:**
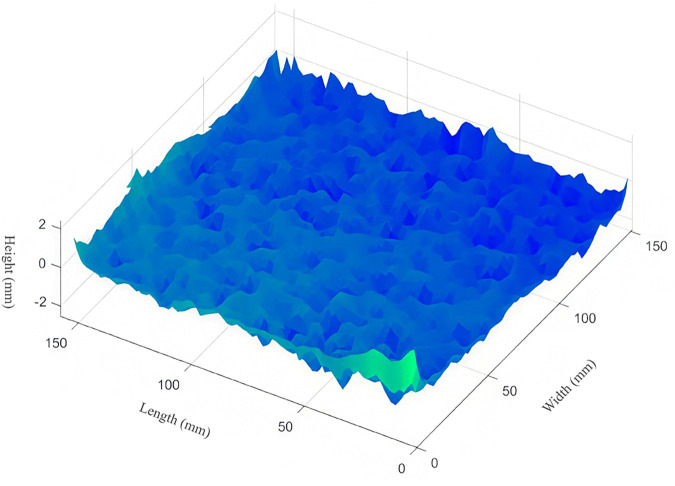
Three-dimensional reconstruction results of the asphalt pavement surface.

A Delaunay triangulation mesh was constructed for the sparse pavement texture point cloud data through linear interpolation, generating a three-dimensional image of the asphalt pavement texture point cloud, as shown in Fig 6.

#### 3.1.4. Acquiring binary images through reverse engineering.

Select the top-down view of the surface layer from the three-dimensional reconstruction model of the road surface for binarization processing. This serves as the standard image for training the neural network model. The specific operational steps are as follows:

(1) Extract the top-down view of the surface layer from the three-dimensional reverse-engineered road texture model through MATLAB programming.
(2) Convert the acquired top-down view of the road surface texture into a grayscale image. Process the acquired top-down view using equation (16) to remove hue and saturation while preserving brightness, then convert it to grayscale.

$$\begin{array}{c} f\left(x,y\right)=\sqrt[{2.2}]{0.2973R^{2.2}+0.6274G^{2.2}+0.0753B^{2.2}} \end{array}$$ (16)

(3) Then, a local adaptive thresholding method is employed to binarize the grayscale image. This approach calculates dynamic thresholds based on the grayscale characteristics of local image regions, effectively mitigating the impact of uneven illumination on segmentation results compared to global thresholding methods.

**Fig 7:**
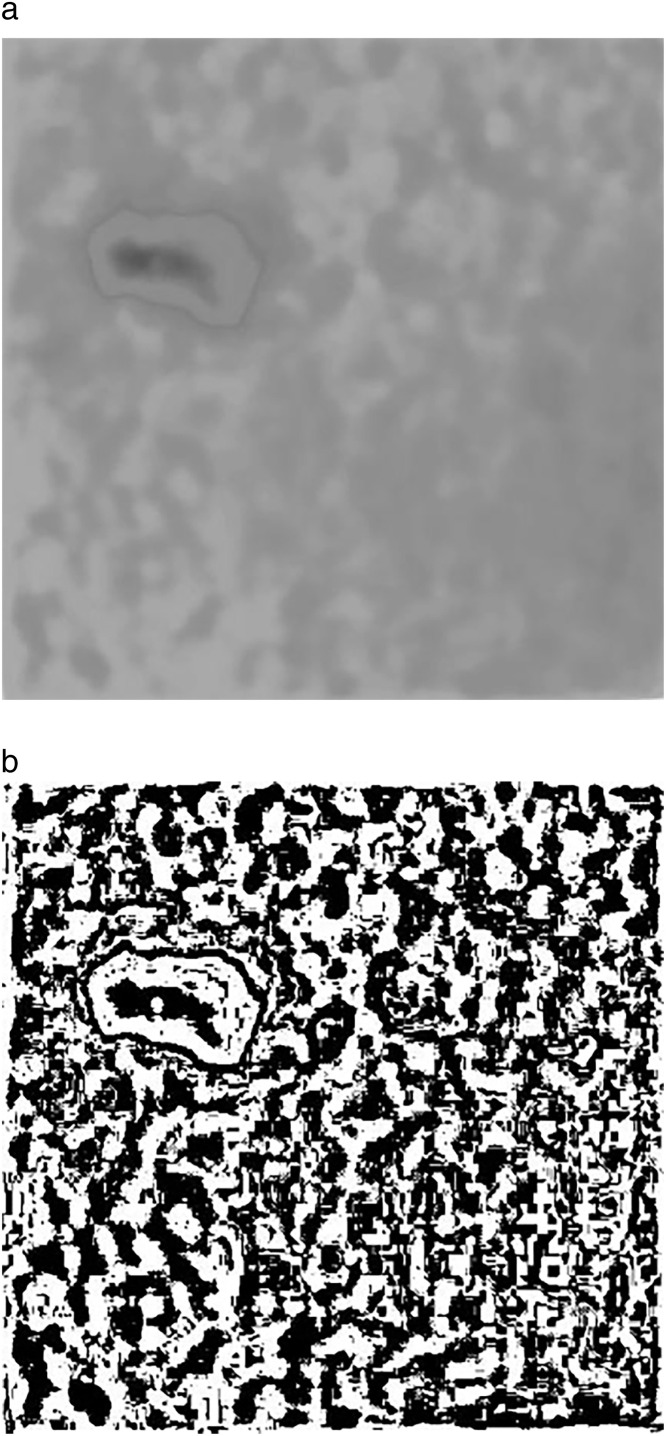
Reversely acquired binary map. (a) Grayscale image to be processed. (b) Binarized result image.

Adaptive threshold segmentation primarily encompasses two forms: mean-adaptive segmentation and weight-adaptive segmentation. This paper employs weighted adaptive segmentation. Within an odd-sized square template window, it performs Gaussian-weighted summation of neighboring pixel grayscale values to compute a local dynamic threshold, thereby converting the grayscale image into a binary image. This method mitigates segmentation errors caused by brightness variations and enhances the accuracy of identifying boundaries between aggregates and voids.. The outcome is illustrated in Fig 7.

### 3.2. Asphalt Pavement Image Segmentation Based on the U-Net Model

**Fig 8:**
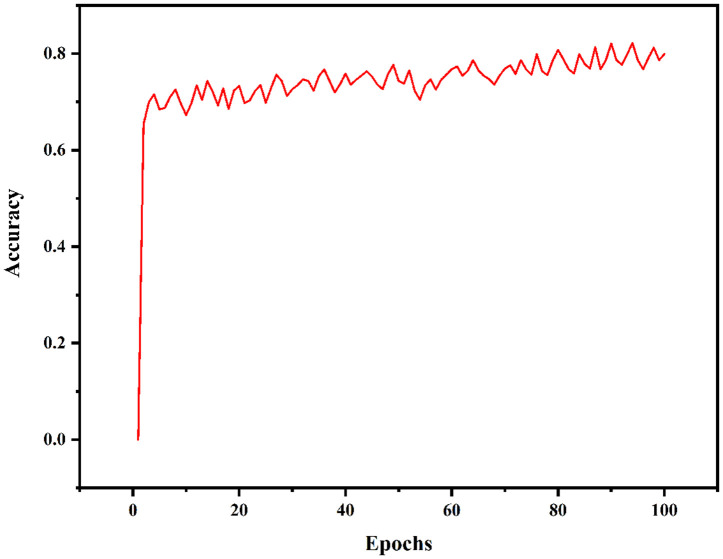
Model training phase accuracy.

**Fig 9:**
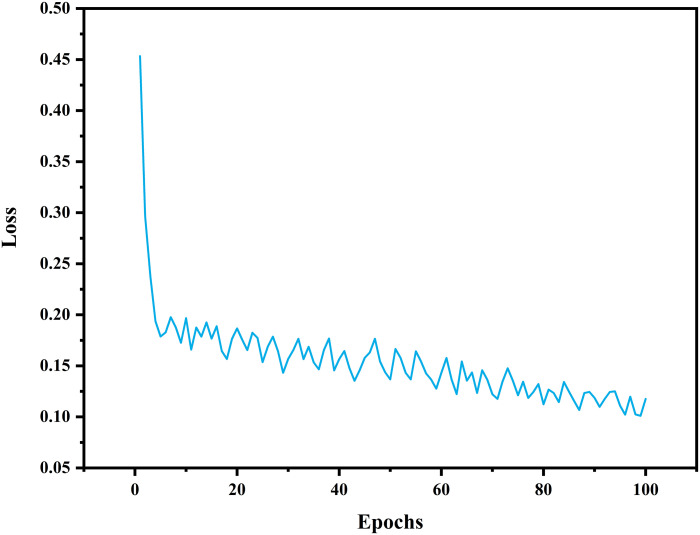
Model training phase loss.

The model accuracy and loss function during the training process are shown in Figs 8 and 9.

Applying the trained model to the validation set yielded evaluation metric results as shown in Table 2.

**Table 2:** Test Set Result.

Network model	A(accuracy)	P(precision)	R(recall)	F1(F1-score)	IoU(Intersection over Union)
U-Net	0.7823	0.8065	0.6928	0.7214	0.5642

According to Table 2, the performance metrics of the trained U-Net model on the test set include an accuracy of 0.7823, a precision of 0.8065, a recall of 0.6928, an F1-score of 0.7214, and an Intersection over Union (IoU) value of 0.5642. These results indicate that the proposed model achieves acceptable performance in distinguishing pavement aggregates and void regions in asphalt pavement images under the investigated conditions.

Although certain segmentation errors still exist in regions with shadows or complex texture boundaries, the generated binary images were still able to support subsequent texture parameter calculation and correlation analysis with satisfactory consistency. The extracted texture parameters maintained good agreement with the measured values obtained using the sand patch method, demonstrating the applicability of the proposed method for pavement texture characterization.

**Fig 10:**
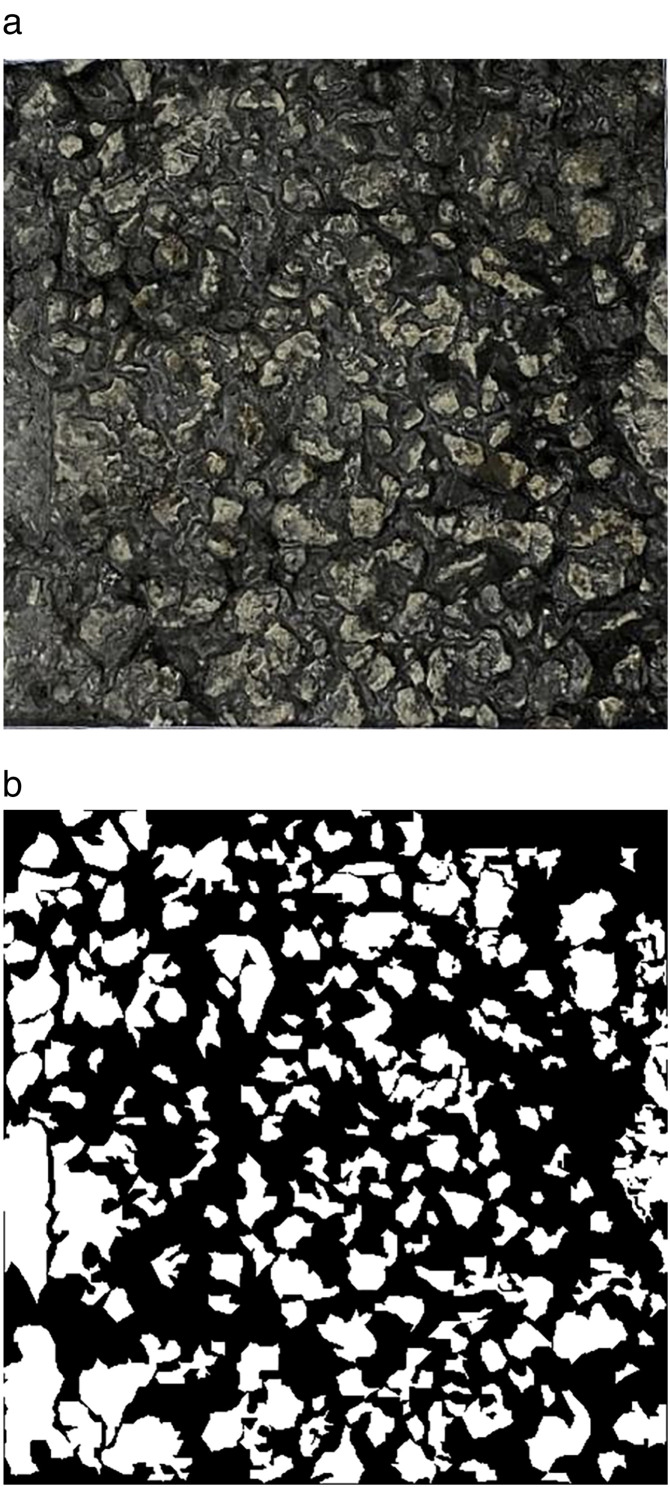
Image segmentation results. (a) Asphalt pavement image. (b) Segmentation results.

Images not used in training the U-Net model were selected for validation. Fig 10 shows asphalt pavement images and their segmentation results.

### 3.3. Accuracy of skid resistance prediction for asphalt pavement

#### 3.3.1. Calculation of mean texture depth.

Thirty-two test points were randomly selected for the test set. The structural depth of the binary image road surface texture processed by deep learning segmentation and the structural depth measured by the sand patch method were calculated. The results are shown in Table 3.

**Table 3:** Comparative analysis of texture depth calculated from binary images and measured by the sand patch method.

Test point number	Sand patch method MTD-1(mm)	Binary image MTD-2(mm)	Absolute error (mm)	Relative error
1	0.73326	0.7162	0.01706	0.02327
2	0.73412	0.6816	0.05252	0.07154
3	0.72892	0.7014	0.02752	0.03775
4	0.73029	0.6913	0.03899	0.05339
5	0.7336	0.6823	0.0513	0.06993
6	0.72248	0.6511	0.07138	0.0988
7	0.72019	0.7057	0.01449	0.02012
8	0.7141	0.6248	0.0893	0.12505
9	0.70988	0.6854	0.02448	0.03448
10	0.41794	0.512	0.09406	0.225056
11	0.34228	0.4701	0.12782	0.373437
12	0.3458	0.5232	0.1774	0.513013
13	0.35862	0.5244	0.16578	0.462272
14	0.35765	0.4326	0.07495	0.209562
15	0.37239	0.5477	0.17531	0.47077
16	0.38015	0.4874	0.10725	0.282125
17	0.35796	0.541	0.18304	0.511342
18	0.36743	0.4782	0.11077	0.301472
19	0.34186	0.4828	0.14094	0.412274
20	0.70561	0.7442	0.03859	0.05469
21	0.57935	0.6589	0.07955	0.137309
22	0.45407	0.5542	0.10013	0.220517
23	0.65813	0.6175	0.04063	0.06174
24	0.45471	0.527	0.07229	0.15898
25	0.56501	0.6274	0.06239	0.110423
26	0.56983	0.6582	0.08837	0.155081
27	0.67386	0.6814	0.00754	0.011189
28	0.68269	0.6921	0.00941	0.013784
29	0.5658	0.6132	0.0474	0.083775
30	0.46932	0.4879	0.01858	0.039589
31	0.47433	0.5344	0.06007	0.126642
32	0.47376	0.578	0.10424	0.220027

**Fig 11:**
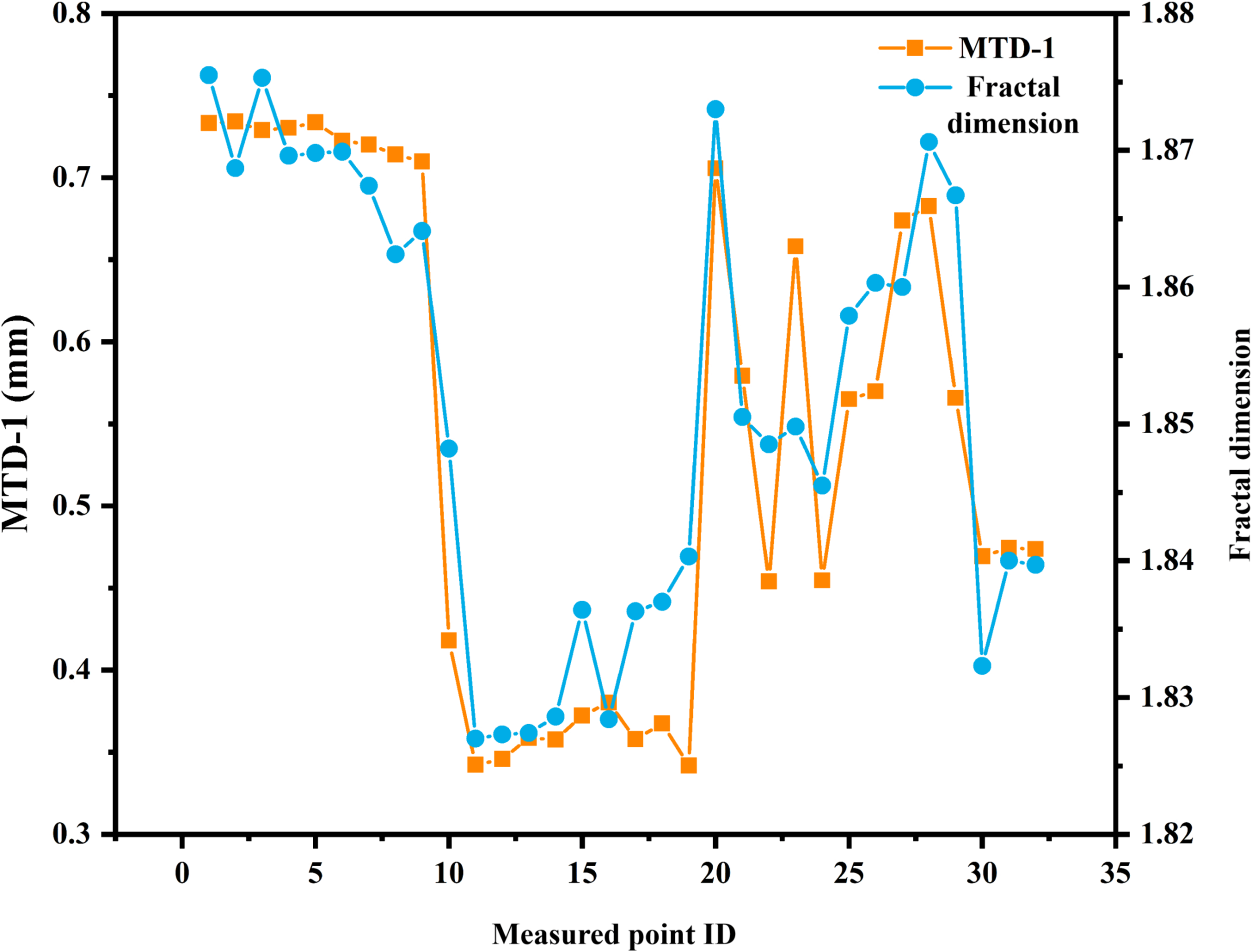
Line graph comparing structural depth calculated from binary images with structural depth measured by the sand patch method.

As shown in Table 3 and Fig 11, after excluding outliers 12, 13, 15, and 17, the maximum absolute error of the binary image calculation results is 0.14094 mm, with an average relative error of around 15%. Among the 28 points after outlier removal, the relative error between 10 measured points and the average structural depth obtained by the sand-laying method does not exceed 5%. while the relative error for 15 measured points did not exceed 10%.

**Fig 12:**
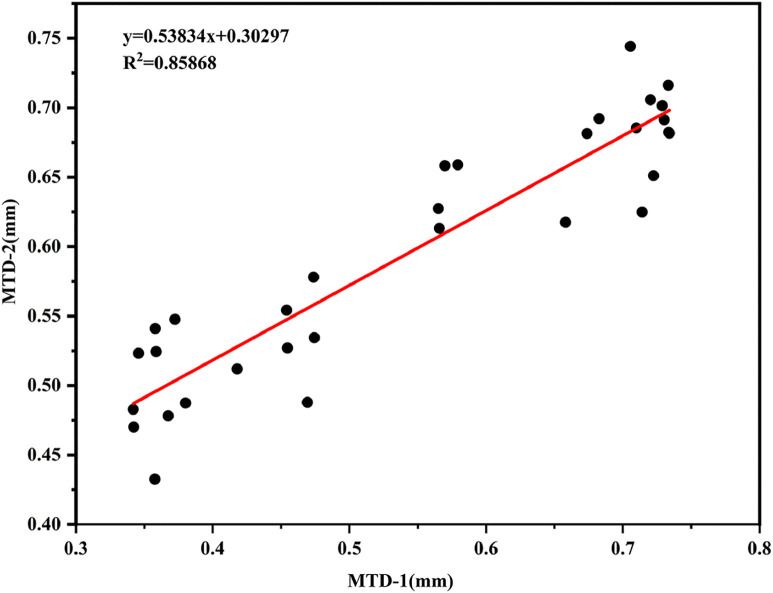
Correlation analysis between MTD-1 and MTD-2.

A correlation analysis was performed between the average constructed depth obtained from the binary image and the data acquired via the sand patch method, as shown in Fig 12.

Comparative analysis reveals that the R² value between MTD-2 derived from binary images and MTD-1 obtained from the sand patch test reaches 0.85, indicating a strong correlation between the two. This suggests that the structural depth predicted by binary images holds certain feasibility in representing the surface structural depth of asphalt pavements.

**Fig 13:**
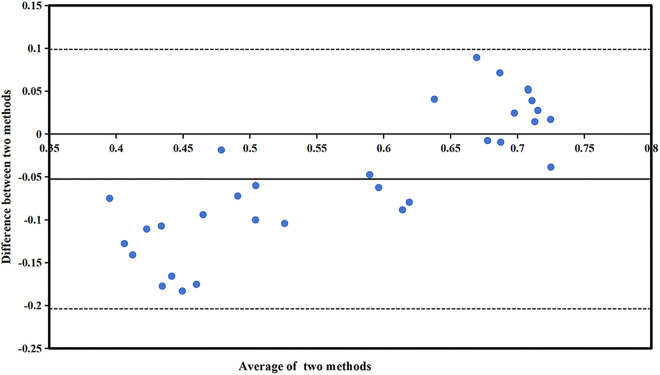
Bland-Altman plot comparing MTD-1 and MTD-2.

To further assess the agreement between the proposed image-based method and the conventional sand block method, a Bland-Altman analysis was performed, as shown in Fig 13. Most data points fall within the 95% confidence limits, and the mean difference between the two methods is relatively small. This indicates that, under the conditions studied, the proposed method maintains acceptable agreement with the sand block method.

Combined with the absolute and relative error analyses presented in Table 3, the results further demonstrate that the proposed image-based method can provide relatively reliable pavement texture depth characterization and support texture-based pavement evaluation.

In addition, the conventional sand patch method adopted in this study was conducted in accordance with the Specification for Quality Inspection and Evaluation of Highway Engineering-Part 1: Civil Engineering Works (JTG F80/1) [43]. The relatively small differences observed between the proposed image-based method and the standard sand patch measurements further suggest that the proposed method can satisfy the requirements for pavement texture evaluation under the investigated conditions.

#### 3.3.2. Calculation of fractal dimension.

The fractal dimension of the binary image of the test road surface was obtained using the calculation method described in Section 2.2.2. The results are shown in Table 4.

**Table 4:** Calculation results of fractal dimensions of asphalt pavement.

Test point	Fractal dimension	Test point	Fractal dimension	Test point	Fractal dimension	Test point	Fractal dimension
1	1.8755	9	1.8641	17	1.8363	25	1.8579
2	1.8687	10	1.8482	18	1.837	26	1.8603
3	1.8753	11	1.827	19	1.8403	27	1.86
4	1.8696	12	1.8273	20	1.873	28	1.8706
5	1.8698	13	1.8274	21	1.8505	29	1.8667
6	1.8699	14	1.8286	22	1.8485	30	1.8323
7	1.8674	15	1.8364	23	1.8498	31	1.84
8	1.8624	16	1.8284	24	1.8455	32	1.8397

**Fig 14:**
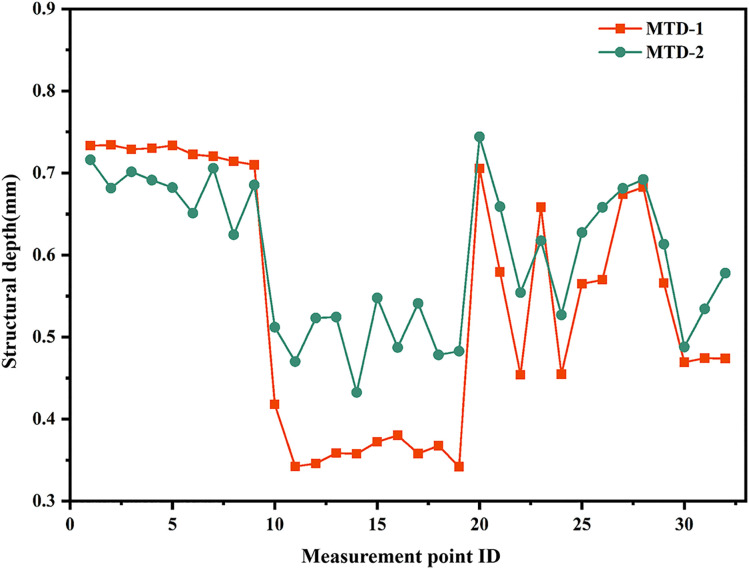
Comparison of fractal dimension and construction depth with MTD-1.

**Fig 15:**
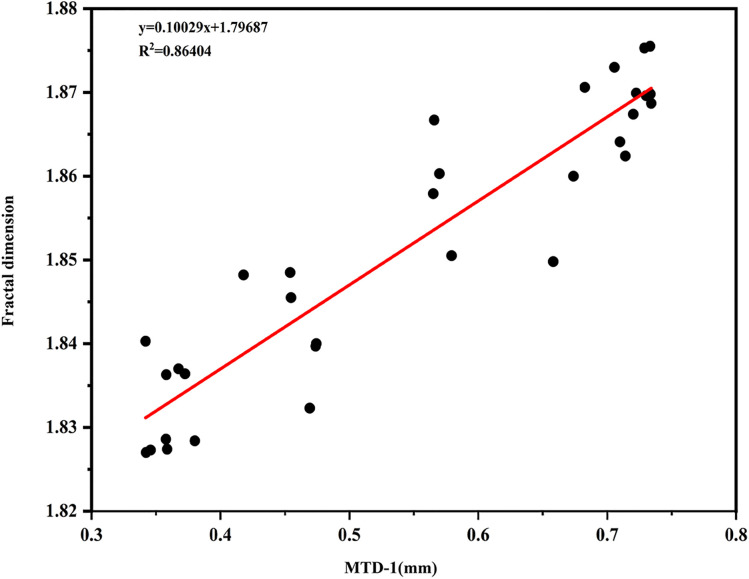
Correlation analysis between fractal dimension and MTD-1.

Based on the fractal dimension calculations in Table 4 and Fig 14, the fractal dimensions of the binary images all fall between 1 and 2. The closer the value is to 2, the higher the complexity of the road surface. The maximum fractal dimension was 1.8755 at Test Point 1, with the minimum value recorded at 1.827 for Test Point 11. The difference between the maximum and minimum values is 0.0485. Further comparison reveals that a higher fractal dimension indicates a rougher road surface and better skid resistance. To further analyze surface texture characteristics, a correlation analysis was conducted between the calculated fractal dimensions and the textural depth (MTD-1) measured using the sand patch method. The results are shown in Fig 15.

Comparative analysis reveals that the correlation coefficient R² between fractal dimension derived from binary images and MTD-1 data obtained via the sand patch method is 0.86. Pearson correlation analysis further indicated that the relationship was statistically significant (r = 0.93, p < 0.001, 95% CI: 0.83–0.96), indicating a strong correlation between the two.

During the comparative analysis, some samples exhibited significant deviations. As discussed in Sections 3.1.2 and 3.1.4, these outliers primarily stemmed from local uneven illumination, shadow occlusion, or artifacts generated during SFM reconstruction, which amplified errors in local elevation extraction. Considering these errors stem from environmental disturbances during imaging and reconstruction rather than systematic biases in the model prediction itself, a small number of affected samples were excluded from the correlation analysis to more objectively reflect the model’s predictive performance under normal conditions.

After removing outliers, the correlation between the fractal dimension and MTD-1 remained at a high level. This further demonstrates that under normal operating conditions, the fractal dimension extracted using the method described in this paper serves as a viable and accurate pavement texture parameter for characterizing skid resistance performance.

Although the proposed detection method demonstrates strong correlation validation, it retains certain limitations for practical engineering applications and requires further optimization in subsequent research. First, the model exhibits sensitivity to ambient lighting conditions. Under extreme shadowing or strong reflective interference, segmentation accuracy of aggregate edges may fluctuate. Future research will focus on constructing datasets incorporating more extreme scenarios and introducing preprocessing algorithms like Contrast-Limited Adaptive Histogram Equalization (CLAHE) to enhance model robustness. Second, the SFM 3D reconstruction process involves intensive feature matching computations, demanding high hardware performance and limiting its real-time field evaluation capabilities. Future improvements will focus on developing lightweight deep learning architectures like MobileNetV3-UNet and optimizing processing workflows using edge computing devices such as NVIDIA Jetson, enabling rapid response from field data collection to real-time parameter evaluation.

## 4. Discussion

This study discusses dataset diversity and its potential influence on model generalization capability. During dataset construction, images were captured using a CCD industrial camera under varying lighting conditions (e.g., shadow and direct sunlight) and different pavement surface conditions, which helps improve dataset diversity under the investigated scenarios.

Although the proposed U-Net-based pavement binary segmentation and texture parameter calculation method demonstrated satisfactory performance on the current dataset, the relatively limited dataset size may still constrain the generalization capability of the model. In addition, the current model was mainly trained and validated under dry and clean pavement conditions. Therefore, its applicability under different lighting environments (e.g., nighttime or wet conditions), different pavement types (e.g., OGFC and SMA), and different pavement wear stages still requires further validation using larger and more diverse field datasets.

Regarding segmentation error analysis, common errors were mainly observed in the over-segmentation of fine aggregate edges and the under-segmentation of dark void regions. These limitations are primarily associated with image resolution constraints and local artifacts generated during the SFM reconstruction process. Such segmentation deviations may affect void ratio calculations in binary images and introduce minor disturbances to the calculated texture parameters, including MTD and fractal dimension.

Nevertheless, experimental comparisons indicate that the use of 3D ground-truth labels obtained through reverse reconstruction can effectively reduce segmentation errors and maintain good consistency between predicted parameters and measured values under the investigated conditions. In particular, the proposed method achieved good agreement with the conventional sand patch method in both correlation analysis and Bland-Altman analysis, demonstrating its applicability for pavement texture characterization and texture-based pavement evaluation.

In addition, the SFM-based three-dimensional reconstruction process involves relatively intensive feature matching and dense point cloud computation, resulting in comparatively high computational complexity. At present, the proposed method is more suitable for localized and refined pavement texture evaluation scenarios. Future studies should therefore focus on lightweight algorithm optimization and computational efficiency improvement to better support lane-level continuous pavement assessment, potentially combined with edge-computing devices for enhanced real-time processing capability.

Overall, the proposed non-contact pavement texture extraction framework demonstrates potential for pavement texture characterization and texture-based pavement evaluation under the investigated conditions. However, broader external validation under more diverse roadway and environmental conditions is still required before large-scale engineering application.

## 5. Conclusions

(1) A model experimental environment was established, and the experimental results were analyzed. After training and testing, the model achieved an accuracy of 0.7823, a precision of 0.8065, a recall of 0.6928, an F1-score of 0.7214, and an IoU value of 0.5642. These results indicate that the proposed model demonstrates acceptable performance in distinguishing aggregate and void regions in asphalt pavement images under the investigated conditions. The generated binary images were able to support subsequent pavement texture parameter extraction and texture-based pavement evaluation with satisfactory consistency.
(2) A method for acquiring inverse binary images based on 3D reconstruction models was proposed. By performing top-down projection and binarization on the surface layer of the 3D reconstructed pavement model, high-precision, low-noise training sample images were generated. This effectively enhanced the training efficiency and segmentation accuracy of the U-Net model, offering a new feasible approach for constructing asphalt pavement image datasets.
(3) For texture parameter extraction, a texture depth calculation method based on gray-level differences was proposed by jointly processing U-Net-segmented binary images and grayscale images. Results show that the average relative error between the texture depth MTD-2 calculated from binary images and the MTD-1 texture depth measured by the sand patch method is approximately 15%, validating the reliability of the model’s computational results.
(4) Correlation analysis was conducted between the texture parameters extracted from binary images and the MTD-1 values measured using the sand patch method. The obtained correlation coefficients (R²) for the average texture depth and fractal dimension were 0.85 and 0.86, respectively, indicating good consistency between the proposed image-based method and the conventional measurement approach.

The results show that the fractal dimension values of the investigated pavement sections were mainly distributed within the range of 2.1–2.5 and gradually decreased with increasing texture wear, reflecting the evolution of pavement surface complexity and texture degradation. Although the fractal dimension demonstrated sensitivity to variations in pavement texture characteristics related to skid resistance, the quantitative classification of pavement safety levels still requires further validation using friction-related indicators and relevant engineering standards.
