# Correction: Binary image acquisition and texture parameter calculation of asphalt pavement based on a U-Net model

**DOI:** 10.1371/journal.pone.0357118

**Published:** 2026-08-26

**Authors:** Fengwei An, Haoran Jiang, Yulong Zhao, Lei Fang, Shujing Dong, Wenpeng Du, Guohua Wu, Wei Liu, Zhenglong Lv, Hao Liang

There are errors in the author affiliations. The correct affiliations are as follows:

Fengwei An^1,2^, Haoran Jiang^3^, Yulong Zhao^4^, Lei Fang^5^, Shujing Dong^4^, Wenpeng Du^3^, Guohua Wu^4^, Wei Liu^1^, Zhenglong Lv^1^, Hao Liang^4^

**1** JSTI GROUP Co., Ltd., Nanjing, China, **2** National Engineering Research Center for Advanced Road Materials, Nanjing, China, **3** Shandong Pengcheng Road and Bridge Group Co., Ltd., Jining, China, **4** School of Transportation and Civil Engineering, Shandong Jiaotong University, Jinan, China, **5** Jining-Jizhou Expressway Co., Ltd., Jining, China.
