# Supplementary material for: Binary image acquisition and texture parameter calculation of asphalt pavement based on a U-Net model
Source: PLoS One. 2026 Jul 22;21(7):e0354127. doi: 10.1371/journal.pone.0354127 (PMC13390860; doi:10.1371/journal.pone.0354127)
Supplement: Attachment — Submitted filename: PLOS ONE.docx [file pone.0354127.s001.docx]

**Manuscript Number:** PONE-D-25-68223
**Title:** Binary Image Acquisition and Texture Parameter Calculation of Asphalt Pavement Based on a U-Net Model

**Recommendation:** Major Revision

**Comments to the Author:**

Thank you for the opportunity to review this well-conceived and technically relevant study. Your work on integrating U-Net segmentation with 3D reconstruction to assess pavement texture represents a meaningful advance toward automated, image-based skid resistance evaluation. The methodological approach is innovative, and the results demonstrate promising correlations with established measurement techniques.

However, to meet the standards of reproducibility, rigor, and transparency expected by PLOS ONE, several important revisions are required. Below, I outline the key concerns that should be addressed in a revised submission.

**Major Revisions Required:**

1. **Methodological Transparency & Reproducibility**
   - The 3D reconstruction pipeline is currently referenced as “Reference 27,” which is absent from the bibliography. Please provide a complete citation or a concise description of the SFM process, including software, parameters, and validation steps.
   - Specify the exact “local thresholding method” used for binarization (e.g., Otsu, adaptive mean) along with its parameters and justification.
   - Consider adding a workflow diagram to visually summarize the complete pipeline—from image acquisition to texture parameter calculation—to aid reader comprehension.
2. **Data Availability Compliance**
   - The current Data Availability Statement does not fulfill PLOS’s requirements. All underlying data—including raw images, 3D models, binary masks, and relevant code/weights—must be deposited in a public repository (e.g., Figshare, Zenodo) with persistent identifiers (DOIs). Please update the statement accordingly.
3. **Statistical Rigor & Validation**
   - Provide a statistical or reasoned justification for the exclusion of outliers in the MTD comparison. If using a statistical test (e.g., Grubbs’, IQR), report it; otherwise, explain potential causes (e.g., shadows, reconstruction artifacts).
   - Report confidence intervals or p-values for correlation coefficients, and consider including a Bland–Altman plot to assess agreement between methods.
   - Discuss the diversity of the dataset (lighting, pavement type, condition) and any potential biases. Analyze common segmentation errors and their possible impact on texture parameter accuracy.
   - Acknowledge the need for external validation under varied conditions in future work.
4. **Interpretation & Limitations**
   - Discuss the practical significance of the observed fractal dimension range: is it sensitive enough to distinguish between safe and unsafe pavement conditions?
   - Expand the discussion of limitations to include performance under wet/contaminated surfaces, different asphalt mix types, and scalability to lane-level assessment.
   - Briefly outline potential real-world deployment scenarios (e.g., integration into automated survey vehicles) in the conclusion.

**Minor Revisions Suggested:**

- Ensure all referenced figures (e.g., Fig. 10, 11, 13) are included and clearly labeled.
- Justify or cite the uncommon grayscale conversion formula (Equation 15).
- Perform thorough proofreading to correct minor grammatical inconsistencies and hyphenation variations (e.g., “sand-patch” vs. “sand-spreading”).

**Overall Impression:**

This study presents a clever and practical application of deep learning in pavement engineering. With careful attention to the points above particularly those concerning methodological transparency, data sharing, and statistical robustness the manuscript will become a stronger and more reproducible contribution. I am optimistic about the potential impact of this approach and encourage the authors to undertake these revisions.

I look forward to reviewing an improved version of the manuscript.

**Best regards,**
