# Supplementary material for: Binary image acquisition and texture parameter calculation of asphalt pavement based on a U-Net model
Source: PLoS One. 2026 Jul 22;21(7):e0354127. doi: 10.1371/journal.pone.0354127 (PMC13390860; doi:10.1371/journal.pone.0354127)
Supplement: Attachment — Submitted filename: Response to Reviewers.docx [file pone.0354127.s003.docx]

**Response to Reviewers**

First of all, we would like to thank you for reviewing our paper and for the thoughtful comments, which help to improve the quality of our manuscript. Accordingly, we have revised our manuscript, and a lot of changes have been made to the manuscript. Also, the detailed responses to the comments are given below:

**Response to Comments from Journal Requirements**

**Comment 1:**When submitting your revision, we need you to address these additional requirements.

**Response:** Thank you for your guidance regarding the additional requirements for the revision. We have carefully reviewed the journal’s instructions and have revised the manuscript accordingly to ensure full compliance with the submission guidelines.All requested modifications have been implemented in the revised version.

**Comment 2:** In your Methods section, please provide additional information regarding the permits you obtained for the work. Please ensure you have included the full name of the authority that approved the field site access and, if no permits were required, a brief statement explaining why.

**Response:** Thank you for your comment regarding ethical approval and site access permission. The data were collected from publicly accessible road sections. No special permission was required for image acquisition, as no restricted areas were involved and no personal or sensitive information was collected.

**Comment 3:** Please note that PLOS One has specific guidelines on code sharing for submissions in which author-generated code underpins the findings in the manuscript. In these cases, we expect all author-generated code to be made available without restrictions upon publication of the work. Please review our guidelines at [https://journals.plos.org/plosone/s/materials-and-software-sharing#loc-sharing-code](https://journals.plos.org/plosone/s/materials-and-software-sharing#loc-sharing-code) and ensure that your code is shared in a way that follows best practice and facilitates reproducibility and reuse.

**Response:** Thank you for the reminder regarding the code sharing policy. The author-generated scripts used in this study have been archived in the Zenodo repository and are publicly available via DOI [10.5281/zenodo.18870696](https://doi.org/10.5281/zenodo.19175141) to support the reproducibility of the results.

**Comment 4:** Thank you for stating the following financial disclosure:

“This work was supported by the National Engineering Research Center of Advanced Road Materials under Grant 202400000114, the Shandong Provincial Natural Science Foundation under Grant ZR2020QE274, the Key Research and Development Program of Shandong Province under Grants 2020RKB01602 and 2025RKY0204, the Open Project of Shandong Key Laboratory of Highway Technology and Safety Assessment under Grant SH202103, and the Science and Technology Plan of Shandong Transportation Department under Grants 2022B102, 2023B92-01, 2023B25, 2024B67-02, 2025BDK02-02, 2025BDK02-01, and 2025BXH04-01.”

Please state what role the funders took in the study.  If the funders had no role, please state: "The funders had no role in study design, data collection and analysis, decision to publish, or preparation of the manuscript."

If this statement is not correct you must amend it as needed.

Please include this amended Role of Funder statement in your cover letter; we will change the online submission form on your behalf.

**Response:** Thank you for the reminder regarding the Role of the Funder statement. We confirm that the funders had no involvement in the study design, data collection and analysis, decision to publish, or preparation of the manuscript.

**Comment 5:** Please note that funding information should not appear in any section or other areas of your manuscript. We will only publish funding information present in the Funding Statement section of the online submission form. Please remove any funding-related text from the manuscript.

**Response:** Thank you for the clarification. We have revised the manuscript accordingly and removed all funding information from the manuscript file.

**Comment 6:** We note that your Data Availability Statement is currently as follows:

“All relevant data are within the manuscript and its Supporting Information files.”

Please confirm at this time whether or not your submission contains all raw data required to replicate the results of your study. Authors must share the “minimal data set” for their submission. PLOS defines the minimal data set to consist of the data required to replicate all study findings reported in the article, as well as related metadata and methods (https://journals.plos.org/plosone/s/data-availability#loc-minimal-data-set-definition).

For example, authors should submit the following data:

- The values behind the means, standard deviations and other measures reported;

- The values used to build graphs;

- The points extracted from images for analysis.

Authors do not need to submit their entire data set if only a portion of the data was used in the reported study. If your submission does not contain these data, please either upload them as Supporting Information files or deposit them to a stable, public repository and provide us with the relevant URLs, DOIs, or accession numbers. For a list of recommended repositories, please see https://journals.plos.org/plosone/s/recommended-repositories.

If there are ethical or legal restrictions on sharing a de-identified data set, please explain them in detail (e.g., data contain potentially sensitive information, data are owned by a third-party organization, etc.) and who has imposed them (e.g., an ethics committee). Please also provide contact information for a data access committee, ethics committee, or other institutional body to which data requests may be sent. If data are owned by a third party, please indicate how others may request data access.

**Response:** Thank you for the reminder regarding the data availability requirements. We confirm that the minimal dataset required to reproduce the results reported in this study has been provided. The data supporting the findings of this study are publicly available via the following DOI [10.5281/zenodo.18870696](https://doi.org/10.5281/zenodo.19175141).

**Comment 7:** PLOS requires an ORCID iD for the corresponding author in Editorial Manager on papers submitted after December 6th, 2016. Please ensure that you have an ORCID iD and that it is validated in Editorial Manager. To do this, go to ‘Update my Information’ (in the upper left-hand corner of the main menu), and click on the Fetch/Validate link next to the ORCID field. This will take you to the ORCID site and allow you to create a new iD or authenticate a pre-existing iD in Editorial Manager.

**Response:** Thank you for the reminder. The corresponding author has registered and validated the ORCID iD in Editorial Manager as required.

**Comment 8:** Thank you for stating the following in the Financial Disclosure section:

“This work was supported by the National Engineering Research Center of Advanced Road Materials under Grant 202400000114, the Shandong Provincial Natural Science Foundation under Grant ZR2020QE274, the Key Research and Development Program of Shandong Province under Grants 2020RKB01602 and 2025RKY0204, the Open Project of Shandong Key Laboratory of Highway Technology and Safety Assessment under Grant SH202103, and the Science and Technology Plan of Shandong Transportation Department under Grants 2022B102, 2023B92-01, 2023B25, 2024B67-02, 2025BDK02-02, 2025BDK02-01, and 2025BXH04-01.”

We note that one or more of the authors are employed by a commercial company:

China Communications Construction Jiangsu Highway Engineering Co., Ltd

Shandong Pengcheng Road and Bridge Group Co., Ltd.,

Jining-Jizhou Expressway Co., Ltd.,

1. Please provide an amended Funding Statement declaring this commercial affiliation, as well as a statement regarding the Role of Funders in your study. If the funding organization did not play a role in the study design, data collection and analysis, decision to publish, or preparation of the manuscript and only provided financial support in the form of authors' salaries and/or research materials, please review your statements relating to the author contributions, and ensure you have specifically and accurately indicated the role(s) that these authors had in your study. You can update author roles in the Author Contributions section of the online submission form.

Please also include the following statement within your amended Funding Statement.

“The funder provided support in the form of salaries for authors [insert relevant initials], but did not have any additional role in the study design, data collection and analysis, decision to publish, or preparation of the manuscript. The specific roles of these authors are articulated in the ‘author contributions’ section.”

If your commercial affiliation did play a role in your study, please state and explain this role within your updated Funding Statement.

2. Please also provide an updated Competing Interests Statement declaring this commercial affiliation along with any other relevant declarations relating to employment, consultancy, patents, products in development, or marketed products, etc.

Within your Competing Interests Statement, please confirm that this commercial affiliation does not alter your adherence to all PLOS ONE policies on sharing data and materials by including the following statement: "This does not alter our adherence to  PLOS ONE policies on sharing data and materials.” (as detailed online in our guide for authors [http://journals.plos.org/plosone/s/competing-interests](http://journals.plos.org/plosone/s/competing-interests)) . If this adherence statement is not accurate and  there are restrictions on sharing of data and/or materials, please state these. Please note that we cannot proceed with consideration of your article until this information has been declared.

Please include both an updated Funding Statement and Competing Interests Statement in your cover letter. We will change the online submission form on your behalf.

**Response:** Thank you for your comments regarding the disclosure of commercial affiliations. The authors Fengwei An, Wei Liu, and Zhenglong Lv are employed by China Communications Construction Jiangsu Highway Engineering Co., Ltd.; Haoran Jiang and Wenpeng Du are employed by Shandong Pengcheng Road and Bridge Group Co., Ltd.; Yulong Zhao, Shujing Dong, Guohua Wu, and Hao Liang are employed by Shandong Jiaotong University; and Lei Fang is employed by Jining–Jizhou Expressway Co., Ltd.

These affiliations did not influence the study design, data collection and analysis, decision to publish, or preparation of the manuscript. The specific contributions of each author have been clearly described in the “Author Contributions” section. The Competing Interests statement in the manuscript has been revised accordingly.

**Comment 9:** Please ensure that you refer to Figure 1 in your text as, if accepted, production will need this reference to link the reader to the figure.

**Response:** Thank you for the reminder. We have ensured that Figure 2 is explicitly cited in the main text. Specifically, in Section 2.1.1 (Image Acquisition), we added the sentence: “The image acquisition setup for asphalt pavement surfaces is illustrated in Fig 2.” This revision ensures that all figures are properly cited in the text in chronological order.

**Comment 10:** .If the reviewer comments include a recommendation to cite specific previously published works, please review and evaluate these publications to determine whether they are relevant and should be cited. There is no requirement to cite these works unless the editor has indicated otherwise.

**Response:** Thank you for the reminder. We have carefully reviewed the publications suggested by the reviewers and evaluated their relevance to our study. Relevant references have been considered and cited where appropriate in the revised manuscript.

**Comment 11:**. Thank you for updating your data availability statement. You note that your data are available within the Supporting Information files, but no such files have been included with your submission. At this time we ask that you please upload your minimal data set as a Supporting Information file, or to a public repository such as Figshare or Dryad.Please also ensure that when you upload your file you include separate captions for your supplementary files at the end of your manuscript.As soon as you confirm the location of the data underlying your findings, we will be able to proceed with the review of your submission.

**Response:** Thank you for the guidance regarding the data availability statement. We have now archived the minimal data set supporting our findings in the Zenodo public repository.The materials are publicly accessible via the permanent DOI: 10.5281/zenodo.18870696. In accordance with your request, we have also added a 'Supporting Information' section at the end of the revised manuscript, providing clear captions and descriptions for these supplementary materials.

**Comment 12:**. It appears that your ORCiD iD has not been validated in your Editorial Manager account and we are unable to proceed until that step is complete.

**Response:** Thank you for the notification. We have now accessed the Editorial Manager account and successfully validated the ORCiD iD. We have confirmed that the green iD icon is now associated with the author profile as required. We apologize for the oversight and hope the submission can now proceed to the next stage.

**Response to Comments from Reviewer #1**

Your work on integrating U-Net segmentation with 3D reconstruction to assess pavement texture represents a meaningful advance toward automated, image-based skid resistance evaluation. The methodological approach is innovative, and the results demonstrate promising correlations with established measurement techniques.However, to meet the standards of reproducibility, rigor, and transparency expected by PLOS ONE, several important revisions are required. Below, I outline the key concerns that should be addressed in a revised submission.

**Response:Thank you very much for your comments. We will do our best to improve the quality of the manuscript. Accordingly, the manuscript has been revised.**

1. Methodological Transparency & Reproducibility

**Comment 1:** The 3D reconstruction pipeline is currently referenced as “Reference 27,” which is absent from the bibliography. Please provide a complete citation or a concise description of the SFM process, including software, parameters, and validation steps.

**Response:** Thank you for your valuable suggestion. To address your comment, we have supplemented the complete bibliographic information of Reference 30 and added a concise description of the SFM-based three-dimensional reconstruction procedure in the revised manuscript.Specifically, we now clarify the multi-view feature extraction and matching process, camera pose estimation, sparse and dense point cloud generation, mesh modeling, and texture mapping steps. In addition, the software platform (Agisoft Metashape), the SIFT-based feature matching method, and the validation procedures including scale calibration and model flatness testing have been explicitly stated.These additions enhance the methodological transparency and improve the reproducibility of the study.

References:

[30] DONG S J. Research on anti-skid performance detection and evaluation of asphalt pavement by integrating machine vision and deep learning [D]. Shandong Jiaotong University, 2025. <https://doi.org/10.27864/d.cnki.gsjtd.2025.000140.>

**Comment 2:** Specify the exact “local thresholding method” used for binarization (e.g., Otsu, adaptive mean) along with its parameters and justification.

**Response:** Thank you very much for the insightful suggestion. Accordingly, the manuscript has been revised.To address your comment, we have clarified the specific local thresholding method adopted in the reverse generation of reference binary images. The revised manuscript now explicitly states that a Gaussian-weighted adaptive local thresholding method was employed. In addition, we have supplemented a concise description of the implementation process, including extraction of the top-view image from the reconstructed three-dimensional pavement model, grayscale conversion using MATLAB, and adaptive threshold determination based on regional luminance distribution. The rationale for selecting this method has also been clarified, emphasizing its effectiveness in mitigating uneven illumination effects and improving the accurate delineation of aggregate and void boundaries.This revision enhances the methodological transparency and strengthens the technical rigor of the study.

**Comment 3:** Consider adding a workflow diagram to visually summarize the complete pipeline—from image acquisition to texture parameter calculation—to aid reader comprehension.

**Response:** Thank you very much for the insightful suggestion. Accordingly, the manuscript has been revised. To address your comment, we have added a workflow diagram (Fig. 1) in the Method section to clearly illustrate the overall technical procedure, including image acquisition, preprocessing, SFM-based three-dimensional reconstruction, reference binary image generation, U-Net training and segmentation, texture parameter calculation, and statistical analysis. This addition provides a clearer visualization of the research framework and improves the readability and structural clarity of the manuscript.

**Comment 4:** Data Availability Compliance

o The current Data Availability Statement does not fulfill PLOS’s requirements. All underlying data—including raw images, 3D models, binary masks, and relevant code/weights—must be deposited in a public repository (e.g., Figshare, Zenodo) with persistent identifiers (DOIs). Please update the statement accordingly.

**Response:** Thank you for your suggestion. The relevant raw data and supporting materials have now been organized and deposited in the Zenodo repository. The dataset has been assigned a permanent Digital Object Identifier (DOI): [10.5281/zenodo.18870696](https://doi.org/10.5281/zenodo.19175141). The data can be accessed and downloaded through this link to ensure the reproducibility of the research results.

**Comment 5:** Report confidence intervals or p-values for correlation coefficients, and consider including a Bland–Altman plot to assess agreement between methods.

**Response:** Thank you for this insightful suggestion regarding the statistical depth of our analysis.Regarding the confidence intervals and p-values: In this study, we primarily utilized the coefficient of determination (R^2^) to evaluate the correlation between the proposed U-Net based method and the traditional sand patch method. With R^2^ values reaching 0.85 and 0.86 for MTD and fractal dimension respectively (based on 32 test points), the results clearly demonstrate a strong linear relationship. Given the experimental nature of pavement texture measurements, these R^2^ values are widely accepted in the field as a robust indicator of consistency. Regarding the Bland-Altman plot: While we appreciate the suggestion, we have already provided a detailed quantitative consistency analysis in Table 3, which includes absolute errors and relative errors for each sample point. This direct error analysis effectively evaluates the agreement between the two methods, serving a similar purpose to a Bland-Altman plot. To maintain the conciseness of the manuscript and stay focused on the core algorithmic contributions, we have retained the current error analysis and R^2^ representation as the primary evidence for methodological consistency.

**Comment 6:** Discuss the diversity of the dataset (lighting, pavement type, condition) and any potential biases. Analyze common segmentation errors and their possible impact on texture parameter accuracy.

**Response:** Thank you very much for the insightful suggestion. Accordingly, the manuscript has been revised. To address your comment regarding dataset diversity and model generalization, we have added a new subsection entitled “Dataset Diversity and Segmentation Error Analysis” in the revised manuscript. Specifically, we now clarify that pavement images were collected using a CCD industrial camera under varying illumination conditions (e.g., shadow and direct sunlight) and different pavement surface states to enhance the adaptability and generalization ability of the U-Net model. This design helps mitigate potential bias caused by a single acquisition environment.
Furthermore, we have supplemented an analysis of common segmentation errors, including over-segmentation along fine aggregate boundaries and under-segmentation in dark void regions. These errors are mainly associated with image resolution limitations and local artifacts introduced during the SFM reconstruction process. Their potential influence on void ratio estimation and subsequent texture parameter calculations (e.g., MTD and fractal dimension) has also been discussed. The results demonstrate that training with reverse-generated 3D ground-truth labels effectively reduces segmentation deviations and maintains strong agreement between predicted and measured parameters, confirming the robustness of the proposed method under diverse pavement conditions. These additions strengthen the discussion on model generalization and improve the overall rigor of the study.

**Comment 7:** Acknowledge the need for external validation under varied conditions in future work.

**Response:** Thank you very much for the insightful suggestion. Accordingly, the manuscript has been revised. To address your comment regarding the limitations of dataset diversity and external validation, we have explicitly acknowledged this limitation in the revised manuscript. The discussion now clarifies that although the proposed U-Net-based binary segmentation and texture parameter calculation method demonstrates high accuracy on laboratory and selected field datasets, further validation under more diverse conditions is necessary. We have therefore added a statement outlining future research directions, including validation under different illumination environments (e.g., nighttime and post-rain wet conditions), various pavement types (such as OGFC and SMA), and different levels of surface wear. These additions more clearly define the scope of the current study and highlight the need for broader external validation to enhance model robustness and generalization capability under diverse engineering conditions.

**Comment 8:** Interpretation & Limitations

Discuss the practical significance of the observed fractal dimension range: is it sensitive enough to distinguish between safe and unsafe pavement conditions?

**Response:** Thank you very much for the insightful suggestion. Accordingly, the manuscript has been revised. To address your comment regarding the engineering interpretation of fractal dimension and its practical implications, we have supplemented a more detailed discussion in the revised manuscript. Specifically, we now report that the coefficients of determination (R^2^) between the binary-image-derived parameters and the measured MTD-1 are 0.85 (for average texture depth) and 0.86 (for fractal dimension), respectively, indicating good accuracy and consistency of the proposed method. In addition, the distribution range of fractal dimension (2.1–2.5) has been clarified, and its decreasing trend with increasing texture wear has been analyzed to demonstrate its sensitivity in characterizing pavement microstructural complexity and degradation. At the same time, we explicitly state that although fractal dimension effectively reflects skid resistance variation trends, the quantitative classification of “safe” and “dangerous” conditions still requires further validation in combination with friction coefficient indicators and relevant engineering standards. These additions strengthen the engineering interpretation of the results and clarify the practical applicability and limitations of the proposed parameter.

**Comment 9:** Expand the discussion of limitations to include performance under wet/contaminated surfaces, different asphalt mix types, and scalability to lane-level assessment.

**Response:** Thank you very much for the valuable suggestion. Accordingly, the manuscript has been revised. To address your comment regarding the applicability of the proposed method under complex service conditions and its potential for engineering implementation, we have expanded the “Conclusions and Future Work” section in the revised manuscript. Specifically, the discussion now includes:

(1) the potential impact of wet, contaminated, and varying illumination conditions on model generalization performance;

(2) the applicability of the proposed method to different asphalt mixture types (e.g., OGFC and SMA) with distinct texture morphologies;

(3) the computational complexity of SFM-based 3D reconstruction and its limitations for lane-level continuous rapid evaluation, along with possible directions for lightweight optimization in future work. These additions further clarify the practical applicability, limitations, and future development directions of the proposed approach.

**Comment 10:** Briefly outline potential real-world deployment scenarios (e.g., integration into automated survey vehicles) in the conclusion.

**Response:** Thank you very much for the valuable suggestion. Accordingly, the manuscript has been revised. To address your comment regarding the practical application of the proposed method, we have supplemented the Conclusion section with a brief discussion of its potential engineering implementation. Specifically, we now clarify that the proposed approach can be integrated into automated inspection vehicles to enable non-contact monitoring of pavement skid resistance performance. This addition highlights the practical relevance and potential for real-world deployment of the proposed methodology.

**Comment 11:** Ensure all referenced figures (e.g., Fig. 10, 11, 13) are included and clearly labeled.

**Response:** Thank you for your careful comment. Accordingly, the manuscript has been revised. All referenced figures (e.g., Figs. 10, 11, and 13) have been carefully checked to ensure they are fully presented and clearly labeled in the revised manuscript. This revision improves the completeness and clarity of the figure presentation.

**Comment 12:** Justify or cite the uncommon grayscale conversion formula (Equation 15).

**Response:** Thank you very much for the valuable suggestion. Accordingly, the manuscript has been revised. To address your comment regarding the non-standard grayscale conversion formula (Equation 15), we have provided a clear explanation of its formulation and supplemented the corresponding reference to justify its use. This revision clarifies the theoretical basis of the equation and enhances the methodological rigor of the manuscript.

**Comment 13:** Perform thorough proofreading to correct minor grammatical inconsistencies and hyphenation variations (e.g., “sand-patch” vs. “sand-spreading”).

**Response:** Thank you for your careful comment. Accordingly, the manuscript has been revised. The terminology has been standardized throughout the manuscript, and all references have been unified as “sand patch method.” Previously inconsistent expressions such as “sand-spreading” and “sand-patch” have been corrected. This revision improves the clarity and consistency of the terminology used in the paper.

**Response to Comments from Reviewer #2**

**Comment 1:** All the figures must be centered and their resolution must be improved. Also, add the legends to the figures.

**Response:** Thank you for your valuable suggestion. We have carefully reviewed all the figures and their corresponding legends in the revised manuscript. All figures have been reformatted to ensure they are centered and presented clearly. After re-examination, we confirm that the existing legends already provide complete and sufficient descriptions of the figure contents, symbols, and annotations. Therefore, no additional legend information was added. These adjustments improve the overall presentation quality and consistency of the manuscript.

**Comment 2:** Discuss the novelty of the proposed method in more detail.

**Response:** To address the limitations of existing skid resistance evaluation methods, this study proposes an approach that integrates three-dimensional reconstruction and deep learning for asphalt pavement texture assessment. Multi-view images are processed using the Structure from Motion (SFM) algorithm to generate a geometrically accurate 3D pavement model, overcoming the lack of depth information in conventional 2D image methods.A reverse-mapping strategy is then employed to extract binary images from the 3D model as reference labels for U-Net training, reducing manual labeling bias and improving segmentation reliability. The trained U-Net model automatically identifies aggregates and voids, enabling the calculation of key texture parameters such as mean texture depth (MTD) and fractal dimension.The proposed method is validated through comparison with sand patch method measurements, demonstrating its effectiveness for non-contact pavement texture evaluation.

**Comment 3:** Expand the literature survey by discussing intelligent models available for the analysis of similar kinds of problems, such as A novel feed-through Elman neural network for predicting the compressive and flexural strengths of eco-friendly jarosite mixed concrete: design, simulation and a comparative study, Recurrent neural network-based prediction of compressive and flexural strength of steel slag mixed concrete, etc

**Response:** Thank you very much for the valuable suggestion. Accordingly, the manuscript has been revised. The literature review has been expanded to incorporate additional intelligent models, including ANN, RF, SVR, XGBoost, and other deep learning approaches applied to similar engineering prediction problems. This revision provides a more comprehensive overview of existing intelligent modeling techniques and better positions the present study within the current research landscape.

**Comment 4:** Include the limitations of the proposed strategy and the ways to address them.

**Response:** Thank you for your thoughtful comments. We agree that the practical limitations and future improvement directions of the proposed method should be more clearly articulated. Therefore, we have revised the manuscript to better clarify these aspects. Although the proposed U-Net and 3D reconstruction-based approach demonstrates high accuracy, certain limitations remain for real-world applications. Specifically, the model shows sensitivity to extreme illumination conditions, such as strong shadows or reflections, which may affect segmentation accuracy near aggregate boundaries. In addition, the SFM-based 3D reconstruction process involves computationally intensive feature matching, leading to relatively high hardware requirements and limiting real-time field implementation. To address these issues, we have added a discussion outlining future work, including the construction of more diverse datasets under complex environmental conditions, the incorporation of preprocessing techniques such as CLAHE to improve robustness, and the exploration of lightweight architectures (e.g., MobileNetV3-UNet) combined with edge-computing deployment strategies to enhance on-site efficiency. The above content has been incorporated into the revised manuscript to better clarify the scope, limitations, and future development of the proposed method.

**Comment 5:** Include a future study section and describe it briefly.

**Response:** Thank you for your thoughtful comment. We agree that the limitations related to dataset diversity and external validation should be more clearly stated. Accordingly, the manuscript has been revised. Although the proposed U-Net-based binary segmentation and texture parameter extraction method demonstrates high accuracy on laboratory and selected field datasets, limitations remain regarding dataset diversity. We have therefore clarified this point in the revised manuscript and outlined future work directions. Specifically, future research will focus on broader external validation under more diverse conditions, including different illumination environments (e.g., nighttime and post-rain wet conditions), various pavement types (such as OGFC and SMA), and different levels of surface wear. These efforts aim to further optimize the model and enhance its robustness and generalization capability under diverse engineering scenarios.

**Response to Comments from Reviewer #3**

**Comment 1:** The Abstract does not report quantitative performance metrics or validation results, even though they are presented in the main manuscript. The authors should revise the Abstract to include the most relevant quantitative evaluation outcomes.

**Response:** Thank you for your thoughtful comments. We agree that the abstract should more clearly present the key quantitative performance indicators and validation results of the proposed method. Therefore, we have revised the abstract to explicitly incorporate the most relevant evaluation metrics. Specifically, we have added the F1-score of the U-Net model on the validation set, as well as the coefficients of determination (R^2^) between the calculated texture parameters and the measured MTD values. These quantitative results more directly demonstrate the segmentation accuracy and the consistency between the proposed method and the traditional sand patch measurements. The above modifications have been incorporated into the revised manuscript to enhance the clarity and completeness of the abstract.

**Comment 2:** The authors should reduce redundancy, clearly specify the photogrammetric software and the multi-view reconstruction approach used, and remove any references to automatic translation tools, which are inappropriate for a scientific manuscript.

**Response:** Thank you for your valuable suggestion. We agree that the relevant section required further refinement and clarification. Accordingly, the manuscript has been revised. Specifically, redundant descriptions have been removed to improve conciseness, and additional details regarding the photogrammetry software and multi-view reconstruction method have been explicitly provided. The revised manuscript now clearly states that Agisoft Metashape was used to perform three-dimensional reconstruction based on the Structure from Motion (SFM) algorithm, along with a description of the corresponding workflow. These revisions enhance the clarity and methodological transparency of the study.

**Comment 3:** The Loss function is incorrectly defined. The equation lacks the negative sign required for a proper loss formulation, and the normalization term is ambiguously denoted as “O” rather than the standard sample size . The authors must correct the equation and clearly define all variables to avoid conceptual and implementation ambiguity.

**Response:** Thank you for your careful review and for pointing out the error in the loss function expression. We agree that the original formulation contained inaccuracies that required correction. Specifically, the negative sign in the cross-entropy loss function was missing, and the normalization term was ambiguously denoted by the symbol “O”. The loss function has now been revised to the standard cross-entropy formulation, with the negative sign explicitly included. In addition, the notation has been unified by using N to represent the total number of samples (or pixels), and the corresponding symbol definitions have been clearly provided. These corrections ensure the mathematical accuracy and clarity of the model description in the revised manuscript.
