# Supplementary material for: Binary image acquisition and texture parameter calculation of asphalt pavement based on a U-Net model
Source: PLoS One. 2026 Jul 22;21(7):e0354127. doi: 10.1371/journal.pone.0354127 (PMC13390860; doi:10.1371/journal.pone.0354127)
Supplement: Attachment — Submitted filename: Response_to_Reviewers_auresp_2.docx [file pone.0354127.s004.docx]

**Response to Reviewers**

First of all, we would like to thank you for reviewing our paper and for the thoughtful comments, which help to improve the quality of our manuscript. Accordingly, we have revised our manuscript, and a lot of changes have been made to the manuscript. Also, the detailed responses to the comments are given below:

**Response to Comments from Reviewer #1**

**Comment 1:** The manuscript states that the correlations are statistically significant at the 95% confidence level, but the corresponding p-values, confidence intervals, or statistical test details are not clearly reported. Since the authors rely strongly on correlation-based validation, please provide p-values and/or 95% confidence intervals for the reported R²/correlation results. This would make the statistical interpretation more rigorous and transparent.

**Response:** Thank you very much for the insightful comment. We agree that providing detailed statistical information improves the rigor and transparency of the correlation analysis. Accordingly, the manuscript has been revised to include the statistical test details, corresponding p-values, and 95% confidence intervals for the reported correlation results.

Pearson correlation analysis was performed to evaluate the relationship between the extracted texture parameters and the measured MTD values. The results show that the correlation between fractal dimension and MTD-1 is statistically significant (r = 0.93, R² = 0.86, p < 0.001, 95% CI: 0.83-0.96), confirming the robustness and reliability of the proposed method.

These additions strengthen the statistical interpretation and improve the overall rigor of the manuscript.

**Comment 2:** The response indicates that Table 3 provides absolute and relative errors, but this is not fully equivalent to a formal agreement analysis. The authors may either include a Bland-Altman plot or briefly justify, in the manuscript itself, why the current error analysis is sufficient for evaluating agreement between the proposed image-based method and the sand patch method.

**Response:** Thank you very much for the valuable suggestion. We agree that agreement analysis is important for evaluating the consistency between the proposed image-based method and the conventional sand patch method.

Accordingly, a Bland-Altman analysis has been added in the revised manuscript to further evaluate the agreement between the two methods (Fig 13). The results show that most data points are distributed within the 95% limits of agreement, and the mean difference between the proposed method and the sand patch method remains relatively small, indicating good consistency between the two measurement approaches.

In addition, the absolute and relative error analyses presented in Table 3 further demonstrate that the differences between the proposed method and the standard sand patch method specified in the Quality Inspection and Assessment Standards for Highway Engineering. Volume 1. Civil Engineering (JTG F80/1) are relatively limited and remain within an acceptable range for pavement texture evaluation. These results suggest that the proposed image-based method can provide reliable support for pavement texture characterization under the investigated conditions.

References:

[1] JTG F80/1-2017, Quality Inspection and Assessment Standards for Highway Engineering. Volume 1. Civil Engineering.

Fig 13 Bland-Altman plot comparing MTD-1 and MTD-2

**Comment 3:** The manuscript validates the proposed method mainly against texture-depth-related measures rather than direct friction or skid resistance measurements. Therefore, statements such as “predict the skid resistance of asphalt pavements” should be revised to a more cautious form, such as “support texture-based assessment of pavement skid resistance” unless direct friction coefficient validation is included.

**Response:** Thank you very much for your valuable suggestions. We agree that this study focuses primarily on parameters related to road surface texture, rather than direct measurements of friction coefficients or skid resistance. We have therefore revised the manuscript to use more cautious and accurate wording when describing the assessment of skid resistance.

Specifically, phrases such as ‘predicting skid resistance’ have been amended to ‘supporting skid resistance assessments based on road surface texture’ or ‘reflecting road surface texture characteristics associated with skid resistance’. Furthermore, the discussion section has been revised to explicitly state that the quantitative classification of road safety levels still requires further validation through friction-related indicators and relevant engineering standards.

**Comment 4:** Please verify that the loss function is written in its standard form with correct brackets, negative sign, and all variables clearly defined. At present, the displayed equation appears to have a formatting issue around the logarithmic terms.

**Response:** Thank you very much for the careful review. We agree that the original presentation of the binary cross-entropy loss function contained formatting inconsistencies, including unclear bracket arrangement and notation issues in the logarithmic terms.

Accordingly, the equation has been carefully checked and revised to the standard binary cross-entropy form. The negative sign, bracket structure, and normalization term have been corrected, and all variables involved in the equation are now explicitly defined in the manuscript to improve clarity and readability.

**Comment 5:** The F1-score of 0.7214 indicates acceptable or promising segmentation performance, but the word “accurate” may be slightly strong without further IoU/Dice, confusion matrix, or class-wise performance reporting. Please consider adding IoU/Dice score or a short explanation of why this F1-score is adequate for the intended pavement texture application.

**Response:** Thank you very much for your valuable suggestion. We agree that the interpretation of the segmentation performance should be presented in a more cautious and objective manner. Accordingly, in the revised manuscript, overly strong expressions such as “accurate” have been replaced with more appropriate descriptions, including “effective” or “acceptable.” In addition, following the reviewer’s suggestion, the Intersection over Union (IoU) metric has been added to Table 2, together with the corresponding calculation formula (Equation 6), in order to provide a more comprehensive evaluation of the spatial overlap performance of the model.

Although the obtained F1-score (0.7214) and IoU value (0.5642) indicate moderate pixel-level segmentation performance, this is mainly attributed to the inherent complexity of asphalt pavement textures, irregular void boundaries, and local shadow interference. Nevertheless, the achieved performance is considered sufficient for the intended purpose of this study. It should be emphasized that the primary objective of this work is to support pavement texture parameter extraction and texture-based pavement evaluation, rather than pursuing pixel-level semantic segmentation accuracy alone.

More importantly, although certain segmentation deviations still exist in locally complex regions, the Bland-Altman agreement analysis (Fig. 13) and the strong correlation with the sand patch method (R^2^=0.85 for MTD) indicate that these local pixel-level errors do not significantly affect the reliability of the final texture parameter calculations. Therefore, the current segmentation performance is considered sufficiently reliable to support the proposed pavement texture characterization and evaluation tasks.

The revised Table 2 and the newly added Equation (6) are provided below.

**Table 2 Test Set Result**

| Network model | A(accuracy) | P(precision) | R(recall) | F1(F1-score) | IoU(Intersection over Union) |
| --- | --- | --- | --- | --- | --- |
| U-Net | 0.7823 | 0.8065 | 0.6928 | 0.7214 | 0.5642 |

$\text{IoU}\text{=}\frac{\text{TP}}{\text{TP}\text{+}\text{FP}\text{+}\text{FN}}$	(6)

**Comment 6:** The added discussion on illumination, pavement type, wet/contaminated surfaces, and lane-level scalability is useful. Please ensure that the conclusion does not overgeneralize beyond the available dataset, particularly because external validation under broader road and environmental conditions is still proposed as future work.

**Response:** Thank you very much for the valuable comment. We agree that the conclusions should remain consistent with the scope of the current dataset and experimental conditions.

Accordingly, the manuscript has been revised to avoid overgeneralization regarding the applicability and robustness of the proposed method. More cautious descriptions have been adopted throughout the discussion sections, emphasizing that the current findings were validated only under the investigated pavement and lighting conditions.

In addition, the revised manuscript now further clarifies that broader external validation under different pavement types, lighting conditions, wet or contaminated surfaces, and large-scale roadway environments is still required in future work before wider engineering application can be established.

**Comment 7:** Please proofread the manuscript for spacing, punctuation, equation formatting, figure-caption consistency, and terminology. For example, maintain one consistent term for the reference method, preferably “sand patch method,” throughout the manuscript.

**Response:** Thank you very much for the careful review and valuable suggestions. The manuscript has been thoroughly proofread and revised to improve language clarity and formatting consistency.

Specifically, spacing, punctuation, equation formatting, figure caption consistency, and terminology usage have been carefully checked and corrected throughout the manuscript. In addition, the terminology referring to the reference method has been standardized consistently as “sand patch method” across the entire paper.

**Response to Comments from Reviewer #3**

**Comment 1:** The presentation of the performance metrics (accuracy, precision, recall, and F1-score) should be improved for clarity and consistency. The current text contains minor inconsistencies and redundant phrasing, and it is unclear whether these metrics correspond to training, validation, or test data. A clearer, more structured reporting of these results is recommended, along with a more balanced interpretation of the model's performance.

**Response:** Thank you very much for the valuable suggestion. We agree that the presentation and interpretation of the performance metrics should be clearer and more objective.

Accordingly, the manuscript has been revised to explicitly clarify that the reported accuracy, precision, recall, and F1-score correspond to the test set results. The related descriptions have also been reorganized to improve clarity and consistency while avoiding redundant or overly subjective interpretations of model performance.

**Comment 2:** In addition, the dataset size appears relatively small for training a deep learning model, and its potential impact on generalization should be discussed more explicitly. While the use of cross-validation helps mitigate this limitation, the description of the data partitioning strategy is unclear, as both k-fold cross-validation and a fixed training–validation split (8:2) are mentioned. The authors should clarify this aspect to ensure methodological transparency and reproducibility.

**Response:** Thank you very much for your valuable comments. We agree that a clear description of the data partitioning strategy and a discussion on the dataset scale are essential for the transparency and reproducibility of deep learning research.

In the revised manuscript, we have clarified the data partitioning strategy to eliminate the ambiguity in the previous version. To ensure an objective evaluation of model performance while reducing the risk of overfitting and data leakage, a fixed hold-out validation strategy was adopted [1]. Specifically, a total of 180 asphalt pavement images were randomly divided into training and test sets at a ratio of 8:2 [2], where 144 images were used for model training and the remaining 36 images were reserved as an independent test set for performance evaluation. The test set was not involved during the training process. All images were resized to 512×512 pixels before training.

Regarding the dataset scale, although the number of pavement images is relatively limited compared with large-scale computer vision datasets, the collected images covered different pavement texture and lighting conditions, which helps improve dataset diversity under the investigated conditions. In addition, pavement texture segmentation mainly focuses on aggregate and void feature extraction rather than large-scale object recognition tasks. Previous studies have indicated that, for specialized engineering image segmentation tasks, relatively limited datasets can still provide effective feature representation and stable model performance.

More importantly, despite the relatively limited dataset size, the extracted texture parameters maintained strong correlations with the measured values obtained by the sand patch method, and the Bland–Altman analysis further demonstrated acceptable agreement between the proposed image-based method and the conventional method. These results indicate that the current dataset is sufficient to support pavement texture characterization under the investigated conditions. Nevertheless, broader external validation using larger and more diverse datasets is still required in future work to further evaluate the robustness and generalization capability of the proposed method. The above clarification has been added to the revised manuscript.

References:

[1] Hastie T, Tibshirani R, Friedman J. The Elements of Statistical Learning: Data Mining, Inference, and Prediction. 2nd ed. New York: Springer; 2009.

[2] He M, Lau TL. CrackHAM: A novel automatic crack detection network based on U-Net for asphalt pavement. IEEE Access. 2024;12:12655–12666.

**Comment 3:** Finally, the statistical analysis could be slightly strengthened by including confidence intervals or p-values to support the reported correlations.

**Response:** Thank you very much for the valuable suggestion. We agree that the inclusion of statistical significance indicators can further strengthen the reliability and transparency of the correlation analysis.

Accordingly, Pearson correlation significance analysis has been added in the revised manuscript. The corresponding p-values and 95% confidence intervals are now provided to support the statistical significance of the reported relationships between the extracted texture parameters and the measured MTD values. For example, the correlation analysis between the fractal dimension derived from binary images and the measured MTD values yielded r=0.93, R^2^=0.86, p<0.001, and a 95% confidence interval of 0.83-0.96, indicating a strong and statistically significant correlation.
