# Supplementary material for: Binary image acquisition and texture parameter calculation of asphalt pavement based on a U-Net model
Source: PLoS One. 2026 Jul 22;21(7):e0354127. doi: 10.1371/journal.pone.0354127 (PMC13390860; doi:10.1371/journal.pone.0354127)
Supplement: Peer review history

# Peer review: Decision Letter 0

**DOI:** 10.1371/journal.pone.0354127.r001

**Authors:** Baohua Guo

19 Jan 2026

PONE-D-25-68223

Binary Image Acquisition and Texture Parameter Calculation of Asphalt Pavement Based on a U-Net Model

PLOS One

Dear Dr. Zhao,

Thank you for submitting your manuscript to PLOS ONE. After careful consideration, we feel that it has merit but does not fully meet PLOS ONE’s publication criteria as it currently stands. Therefore, we invite you to submit a revised version of the manuscript that addresses the points raised during the review process.

Please submit your revised manuscript by Mar 05 2026 11:59PM. If you will need more time than this to complete your revisions, please reply to this message or contact the journal office at plosone@plos.org. When you're ready to submit your revision, log on to https://www.editorialmanager.com/pone/ and select the 'Submissions Needing Revision' folder to locate your manuscript file.

Please include the following items when submitting your revised manuscript:

- A letter that responds to each point raised by the academic editor and reviewer(s). You should upload this letter as a separate file labeled 'Response to Reviewers'.
- A marked-up copy of your manuscript that highlights changes made to the original version. You should upload this as a separate file labeled 'Revised Manuscript with Track Changes'.
- An unmarked version of your revised paper without tracked changes. You should upload this as a separate file labeled 'Manuscript'.

If you would like to make changes to your financial disclosure, please include your updated statement in your cover letter. Guidelines for resubmitting your figure files are available below the reviewer comments at the end of this letter.

If applicable, we recommend that you deposit your laboratory protocols in protocols.io to enhance the reproducibility of your results. Protocols.io assigns your protocol its own identifier (DOI) so that it can be cited independently in the future. For instructions see: https://journals.plos.org/plosone/s/submission-guidelines#loc-laboratory-protocols. Additionally, PLOS ONE offers an option for publishing peer-reviewed Lab Protocol articles, which describe protocols hosted on protocols.io. Read more information on sharing protocols at https://plos.org/protocols?utm_medium=editorial-email&utm_source=authorletters&utm_campaign=protocols.

We look forward to receiving your revised manuscript.

Kind regards,

Baohua Guo

Academic Editor

PLOS One

**Journal Requirements:**

When submitting your revision, we need you to address these additional requirements.

1. Please ensure that your manuscript meets PLOS ONE's style requirements, including those for file naming. The PLOS ONE style templates can be found at

https://journals.plos.org/plosone/s/file?id=wjVg/PLOSOne_formatting_sample_main_body.pdf and

https://journals.plos.org/plosone/s/file?id=ba62/PLOSOne_formatting_sample_title_authors_affiliations.pdf

2. In your Methods section, please provide additional information regarding the permits you obtained for the work. Please ensure you have included the full name of the authority that approved the field site access and, if no permits were required, a brief statement explaining why.

3. Please note that PLOS One has specific guidelines on code sharing for submissions in which author-generated code underpins the findings in the manuscript. In these cases, we expect all author-generated code to be made available without restrictions upon publication of the work. Please review our guidelines at https://journals.plos.org/plosone/s/materials-and-software-sharing#loc-sharing-code and ensure that your code is shared in a way that follows best practice and facilitates reproducibility and reuse.

4. Thank you for stating the following financial disclosure:

“This work was supported by the National Engineering Research Center of Advanced Road Materials under Grant 202400000114, the Shandong Provincial Natural Science Foundation under Grant ZR2020QE274, the Key Research and Development Program of Shandong Province under Grants 2020RKB01602 and 2025RKY0204, the Open Project of Shandong Key Laboratory of Highway Technology and Safety Assessment under Grant SH202103, and the Science and Technology Plan of Shandong Transportation Department under Grants 2022B102, 2023B92-01, 2023B25, 2024B67-02, 2025BDK02-02, 2025BDK02-01, and 2025BXH04-01.”

Please state what role the funders took in the study.  If the funders had no role, please state: "The funders had no role in study design, data collection and analysis, decision to publish, or preparation of the manuscript."

If this statement is not correct you must amend it as needed.

Please include this amended Role of Funder statement in your cover letter; we will change the online submission form on your behalf.

5. Please note that funding information should not appear in any section or other areas of your manuscript. We will only publish funding information present in the Funding Statement section of the online submission form. Please remove any funding-related text from the manuscript.

6. We note that your Data Availability Statement is currently as follows:

“All relevant data are within the manuscript and its Supporting Information files.”

Please confirm at this time whether or not your submission contains all raw data required to replicate the results of your study. Authors must share the “minimal data set” for their submission. PLOS defines the minimal data set to consist of the data required to replicate all study findings reported in the article, as well as related metadata and methods (https://journals.plos.org/plosone/s/data-availability#loc-minimal-data-set-definition).

For example, authors should submit the following data:

- The values behind the means, standard deviations and other measures reported;

- The values used to build graphs;

- The points extracted from images for analysis.

Authors do not need to submit their entire data set if only a portion of the data was used in the reported study.

If your submission does not contain these data, please either upload them as Supporting Information files or deposit them to a stable, public repository and provide us with the relevant URLs, DOIs, or accession numbers. For a list of recommended repositories, please see https://journals.plos.org/plosone/s/recommended-repositories.

If there are ethical or legal restrictions on sharing a de-identified data set, please explain them in detail (e.g., data contain potentially sensitive information, data are owned by a third-party organization, etc.) and who has imposed them (e.g., an ethics committee). Please also provide contact information for a data access committee, ethics committee, or other institutional body to which data requests may be sent. If data are owned by a third party, please indicate how others may request data access.

7. PLOS requires an ORCID iD for the corresponding author in Editorial Manager on papers submitted after December 6th, 2016. Please ensure that you have an ORCID iD and that it is validated in Editorial Manager. To do this, go to ‘Update my Information’ (in the upper left-hand corner of the main menu), and click on the Fetch/Validate link next to the ORCID field. This will take you to the ORCID site and allow you to create a new iD or authenticate a pre-existing iD in Editorial Manager.

8. Thank you for stating the following in the Financial Disclosure section:

“This work was supported by the National Engineering Research Center of Advanced Road Materials under Grant 202400000114, the Shandong Provincial Natural Science Foundation under Grant ZR2020QE274, the Key Research and Development Program of Shandong Province under Grants 2020RKB01602 and 2025RKY0204, the Open Project of Shandong Key Laboratory of Highway Technology and Safety Assessment under Grant SH202103, and the Science and Technology Plan of Shandong Transportation Department under Grants 2022B102, 2023B92-01, 2023B25, 2024B67-02, 2025BDK02-02, 2025BDK02-01, and 2025BXH04-01.”

We note that one or more of the authors are employed by a commercial company:

- China Communications Construction Jiangsu Highway Engineering Co., Ltd
- Shandong Pengcheng Road and Bridge Group Co., Ltd.,
- Jining-Jizhou Expressway Co., Ltd.,

1. Please provide an amended Funding Statement declaring this commercial affiliation, as well as a statement regarding the Role of Funders in your study. If the funding organization did not play a role in the study design, data collection and analysis, decision to publish, or preparation of the manuscript and only provided financial support in the form of authors' salaries and/or research materials, please review your statements relating to the author contributions, and ensure you have specifically and accurately indicated the role(s) that these authors had in your study. You can update author roles in the Author Contributions section of the online submission form.

Please also include the following statement within your amended Funding Statement.

“The funder provided support in the form of salaries for authors [insert relevant initials], but did not have any additional role in the study design, data collection and analysis, decision to publish, or preparation of the manuscript. The specific roles of these authors are articulated in the ‘author contributions’ section.”

If your commercial affiliation did play a role in your study, please state and explain this role within your updated Funding Statement.

2. Please also provide an updated Competing Interests Statement declaring this commercial affiliation along with any other relevant declarations relating to employment, consultancy, patents, products in development, or marketed products, etc.

Within your Competing Interests Statement, please confirm that this commercial affiliation does not alter your adherence to all PLOS ONE policies on sharing data and materials by including the following statement: "This does not alter our adherence to  PLOS ONE policies on sharing data and materials.” (as detailed online in our guide for authors http://journals.plos.org/plosone/s/competing-interests) . If this adherence statement is not accurate and  there are restrictions on sharing of data and/or materials, please state these. Please note that we cannot proceed with consideration of your article until this information has been declared.

Please include both an updated Funding Statement and Competing Interests Statement in your cover letter. We will change the online submission form on your behalf.

9. Please ensure that you refer to Figure 1 in your text as, if accepted, production will need this reference to link the reader to the figure.

10. If the reviewer comments include a recommendation to cite specific previously published works, please review and evaluate these publications to determine whether they are relevant and should be cited. There is no requirement to cite these works unless the editor has indicated otherwise.

[Note: HTML markup is below. Please do not edit.]

Reviewers' comments:

Reviewer's Responses to Questions

**Comments to the Author**

1. Is the manuscript technically sound, and do the data support the conclusions?

The manuscript must describe a technically sound piece of scientific research with data that supports the conclusions. Experiments must have been conducted rigorously, with appropriate controls, replication, and sample sizes. The conclusions must be drawn appropriately based on the data presented.

Reviewer #1: Yes

Reviewer #2: Yes

Reviewer #3: Partly

**********

2. Has the statistical analysis been performed appropriately and rigorously?

Reviewer #1: Yes

Reviewer #2: Yes

Reviewer #3: Yes

**********

3. Have the authors made all data underlying the findings in their manuscript fully available?

The PLOS Data policy requires authors to make all data underlying the findings described in their manuscript fully available without restriction, with rare exception (please refer to the Data Availability Statement in the manuscript PDF file). The data should be provided as part of the manuscript or its supporting information, or deposited to a public repository. For example, in addition to summary statistics, the data points behind means, medians and variance measures should be available. If there are restrictions on publicly sharing data—e.g. participant privacy or use of data from a third party—those must be specified.

Reviewer #1: No

Reviewer #2: No

Reviewer #3: No

**********

4. Is the manuscript presented in an intelligible fashion and written in standard English?

PLOS ONE does not copyedit accepted manuscripts, so the language in submitted articles must be clear, correct, and unambiguous. Any typographical or grammatical errors should be corrected at revision, so please note any specific errors here.

Reviewer #1: Yes

Reviewer #2: Yes

Reviewer #3: Yes

**********

5. Review Comments to the Author

Please use the space provided to explain your answers to the questions above. You may also include additional comments for the author, including concerns about dual publication, research ethics, or publication ethics. (Please upload your review as an attachment if it exceeds 20,000 characters)

Reviewer #1: Manuscript Number: PONE-D-25-68223

Title: Binary Image Acquisition and Texture Parameter Calculation of Asphalt Pavement Based on a U-Net Model

Recommendation: Major Revision

Comments to the Author:

Thank you for the opportunity to review this well-conceived and technically relevant study. Your work on integrating U-Net segmentation with 3D reconstruction to assess pavement texture represents a meaningful advance toward automated, image-based skid resistance evaluation. The methodological approach is innovative, and the results demonstrate promising correlations with established measurement techniques.

However, to meet the standards of reproducibility, rigor, and transparency expected by PLOS ONE, several important revisions are required. Below, I outline the key concerns that should be addressed in a revised submission.

Major Revisions Required:

1. Methodological Transparency & Reproducibility

o The 3D reconstruction pipeline is currently referenced as “Reference 27,” which is absent from the bibliography. Please provide a complete citation or a concise description of the SFM process, including software, parameters, and validation steps.

o Specify the exact “local thresholding method” used for binarization (e.g., Otsu, adaptive mean) along with its parameters and justification.

o Consider adding a workflow diagram to visually summarize the complete pipeline—from image acquisition to texture parameter calculation—to aid reader comprehension.

2. Data Availability Compliance

o The current Data Availability Statement does not fulfill PLOS’s requirements. All underlying data—including raw images, 3D models, binary masks, and relevant code/weights—must be deposited in a public repository (e.g., Figshare, Zenodo) with persistent identifiers (DOIs). Please update the statement accordingly.

3. Statistical Rigor & Validation

o Provide a statistical or reasoned justification for the exclusion of outliers in the MTD comparison. If using a statistical test (e.g., Grubbs’, IQR), report it; otherwise, explain potential causes (e.g., shadows, reconstruction artifacts).

o Report confidence intervals or p-values for correlation coefficients, and consider including a Bland–Altman plot to assess agreement between methods.

o Discuss the diversity of the dataset (lighting, pavement type, condition) and any potential biases. Analyze common segmentation errors and their possible impact on texture parameter accuracy.

o Acknowledge the need for external validation under varied conditions in future work.

4. Interpretation & Limitations

o Discuss the practical significance of the observed fractal dimension range: is it sensitive enough to distinguish between safe and unsafe pavement conditions?

o Expand the discussion of limitations to include performance under wet/contaminated surfaces, different asphalt mix types, and scalability to lane-level assessment.

o Briefly outline potential real-world deployment scenarios (e.g., integration into automated survey vehicles) in the conclusion.

Minor Revisions Suggested:

• Ensure all referenced figures (e.g., Fig. 10, 11, 13) are included and clearly labeled.

• Justify or cite the uncommon grayscale conversion formula (Equation 15).

• Perform thorough proofreading to correct minor grammatical inconsistencies and hyphenation variations (e.g., “sand-patch” vs. “sand-spreading”).

Overall Impression:

This study presents a clever and practical application of deep learning in pavement engineering. With careful attention to the points above particularly those concerning methodological transparency, data sharing, and statistical robustness the manuscript will become a stronger and more reproducible contribution. I am optimistic about the potential impact of this approach and encourage the authors to undertake these revisions.

I look forward to reviewing an improved version of the manuscript.

Best regards,

Reviewer #2: 1. All the figures must be centered and their resolution must be improved. Also, add the legends to the figures.

2. Discuss the novelty of the proposed method in more detail.

3. Expand the literature survey by discussing intelligent models available for the analysis of similar kinds of problems, such as A novel feed-through Elman neural network for predicting the compressive and flexural strengths of eco-friendly jarosite mixed concrete: design, simulation and a comparative study, Recurrent neural network-based prediction of compressive and flexural strength of steel slag mixed concrete, etc

4. Include the limitations of the proposed strategy and the ways to address them.

5. Include a future study section and describe it briefly.

Reviewer #3: The Abstract does not report quantitative performance metrics or validation results, even though they are presented in the main manuscript. The authors should revise the Abstract to include the most relevant quantitative evaluation outcomes.

The authors should reduce redundancy, clearly specify the photogrammetric software and the multi-view reconstruction approach used, and remove any references to automatic translation tools, which are inappropriate for a scientific manuscript.

The Loss function is incorrectly defined. The equation lacks the negative sign required for a proper loss formulation, and the normalization term is ambiguously denoted as “O” rather than the standard sample size . The authors must correct the equation and clearly define all variables to avoid conceptual and implementation ambiguity.

The dataset size is limited. While this does not invalidate the study, the authors should explicitly acknowledge this limitation and temper any claims about large-scale generalization or real-world deployment.

The manuscript would benefit from a more structured discussion of the results that critically interprets and contextualizes the findings in relation to the proposed methodology. In addition, Figures 7 and 8 should be improved by including clearer, more informative validation curves, particularly accuracy and loss curves. Enhancing the presentation of these metrics would facilitate a more rigorous interpretation of the training and validation behavior of the proposed model and support a clearer explanation of the reported results.

Overall, the manuscript follows an appropriate structure; however, several sections contain redundant wording and overly long sentences that reduce clarity. A careful stylistic revision is recommended to improve conciseness and precision, particularly in the methodology and conclusions.

Finally, figures illustrating the methodological workflow and the network architectures should be included to enhance clarity and reproducibility.

**********

6. PLOS authors have the option to publish the peer review history of their article (what does this mean?). If published, this will include your full peer review and any attached files.

If you choose “no”, your identity will remain anonymous but your review may still be made public.

**Do you want your identity to be public for this peer review?**  For information about this choice, including consent withdrawal, please see our Privacy Policy.

Reviewer #1: No

Reviewer #2: No

Reviewer #3: No

**********

[NOTE: If reviewer comments were submitted as an attachment file, they will be attached to this email and accessible via the submission site. Please log into your account, locate the manuscript record, and check for the action link "View Attachments". If this link does not appear, there are no attachment files.]

To ensure your figures meet our technical requirements, please review our figure guidelines: https://journals.plos.org/plosone/s/figures

You may also use PLOS’s free figure tool, NAAS, to help you prepare publication quality figures: https://journals.plos.org/plosone/s/figures#loc-tools-for-figure-preparation.

NAAS will assess whether your figures meet our technical requirements by comparing each figure against our figure specifications.

Attachment Submitted filename: PLOS ONE.docx

# Author comment: Author response to Decision Letter 1

**DOI:** 10.1371/journal.pone.0354127.r002

31 Mar 2026

Response to Reviewers

First of all, we would like to thank you for reviewing our paper and for the thoughtful comments, which help to improve the quality of our manuscript. Accordingly, we have revised our manuscript, and a lot of changes have been made to the manuscript. Also, the detailed responses to the comments are given below:

Response to Comments from Journal Requirements

Comment 1:When submitting your revision, we need you to address these additional requirements.

Response: Thank you for your guidance regarding the additional requirements for the revision. We have carefully reviewed the journal’s instructions and have revised the manuscript accordingly to ensure full compliance with the submission guidelines.All requested modifications have been implemented in the revised version.

Comment 2: In your Methods section, please provide additional information regarding the permits you obtained for the work. Please ensure you have included the full name of the authority that approved the field site access and, if no permits were required, a brief statement explaining why.

Response: Thank you for your comment regarding ethical approval and site access permission. The data were collected from publicly accessible road sections. No special permission was required for image acquisition, as no restricted areas were involved and no personal or sensitive information was collected.

Comment 3: Please note that PLOS One has specific guidelines on code sharing for submissions in which author-generated code underpins the findings in the manuscript. In these cases, we expect all author-generated code to be made available without restrictions upon publication of the work. Please review our guidelines at https://journals.plos.org/plosone/s/materials-and-software-sharing#loc-sharing-code and ensure that your code is shared in a way that follows best practice and facilitates reproducibility and reuse.

Response: Thank you for the reminder regarding the code sharing policy. The author-generated scripts used in this study have been archived in the Zenodo repository and are publicly available via DOI 10.5281/zenodo.18870696 to support the reproducibility of the results.

Comment 4: Thank you for stating the following financial disclosure:

“This work was supported by the National Engineering Research Center of Advanced Road Materials under Grant 202400000114, the Shandong Provincial Natural Science Foundation under Grant ZR2020QE274, the Key Research and Development Program of Shandong Province under Grants 2020RKB01602 and 2025RKY0204, the Open Project of Shandong Key Laboratory of Highway Technology and Safety Assessment under Grant SH202103, and the Science and Technology Plan of Shandong Transportation Department under Grants 2022B102, 2023B92-01, 2023B25, 2024B67-02, 2025BDK02-02, 2025BDK02-01, and 2025BXH04-01.”

Please state what role the funders took in the study.  If the funders had no role, please state: "The funders had no role in study design, data collection and analysis, decision to publish, or preparation of the manuscript."

If this statement is not correct you must amend it as needed.

Please include this amended Role of Funder statement in your cover letter; we will change the online submission form on your behalf.

Response: Thank you for the reminder regarding the Role of the Funder statement. We confirm that the funders had no involvement in the study design, data collection and analysis, decision to publish, or preparation of the manuscript.

Comment 5: Please note that funding information should not appear in any section or other areas of your manuscript. We will only publish funding information present in the Funding Statement section of the online submission form. Please remove any funding-related text from the manuscript.

Response: Thank you for the clarification. We have revised the manuscript accordingly and removed all funding information from the manuscript file.

Comment 6: We note that your Data Availability Statement is currently as follows:

“All relevant data are within the manuscript and its Supporting Information files.”

Please confirm at this time whether or not your submission contains all raw data required to replicate the results of your study. Authors must share the “minimal data set” for their submission. PLOS defines the minimal data set to consist of the data required to replicate all study findings reported in the article, as well as related metadata and methods (https://journals.plos.org/plosone/s/data-availability#loc-minimal-data-set-definition).

For example, authors should submit the following data:

- The values behind the means, standard deviations and other measures reported;

- The values used to build graphs;

- The points extracted from images for analysis.

Authors do not need to submit their entire data set if only a portion of the data was used in the reported study. If your submission does not contain these data, please either upload them as Supporting Information files or deposit them to a stable, public repository and provide us with the relevant URLs, DOIs, or accession numbers. For a list of recommended repositories, please see https://journals.plos.org/plosone/s/recommended-repositories.

If there are ethical or legal restrictions on sharing a de-identified data set, please explain them in detail (e.g., data contain potentially sensitive information, data are owned by a third-party organization, etc.) and who has imposed them (e.g., an ethics committee). Please also provide contact information for a data access committee, ethics committee, or other institutional body to which data requests may be sent. If data are owned by a third party, please indicate how others may request data access.

Response: Thank you for the reminder regarding the data availability requirements. We confirm that the minimal dataset required to reproduce the results reported in this study has been provided. The data supporting the findings of this study are publicly available via the following DOI 10.5281/zenodo.18870696.

Comment 7: PLOS requires an ORCID iD for the corresponding author in Editorial Manager on papers submitted after December 6th, 2016. Please ensure that you have an ORCID iD and that it is validated in Editorial Manager. To do this, go to ‘Update my Information’ (in the upper left-hand corner of the main menu), and click on the Fetch/Validate link next to the ORCID field. This will take you to the ORCID site and allow you to create a new iD or authenticate a pre-existing iD in Editorial Manager.

Response: Thank you for the reminder. The corresponding author has registered and validated the ORCID iD in Editorial Manager as required.

Comment 8: Thank you for stating the following in the Financial Disclosure section:

“This work was supported by the National Engineering Research Center of Advanced Road Materials under Grant 202400000114, the Shandong Provincial Natural Science Foundation under Grant ZR2020QE274, the Key Research and Development Program of Shandong Province under Grants 2020RKB01602 and 2025RKY0204, the Open Project of Shandong Key Laboratory of Highway Technology and Safety Assessment under Grant SH202103, and the Science and Technology Plan of Shandong Transportation Department under Grants 2022B102, 2023B92-01, 2023B25, 2024B67-02, 2025BDK02-02, 2025BDK02-01, and 2025BXH04-01.”

We note that one or more of the authors are employed by a commercial company:

China Communications Construction Jiangsu Highway Engineering Co., Ltd

Shandong Pengcheng Road and Bridge Group Co., Ltd.,

Jining-Jizhou Expressway Co., Ltd.,

1. Please provide an amended Funding Statement declaring this commercial affiliation, as well as a statement regarding the Role of Funders in your study. If the funding organization did not play a role in the study design, data collection and analysis, decision to publish, or preparation of the manuscript and only provided financial support in the form of authors' salaries and/or research materials, please review your statements relating to the author contributions, and ensure you have specifically and accurately indicated the role(s) that these authors had in your study. You can update author roles in the Author Contributions section of the online submission form.

Please also include the following statement within your amended Funding Statement.

“The funder provided support in the form of salaries for authors [insert relevant initials], but did not have any additional role in the study design, data collection and analysis, decision to publish, or preparation of the manuscript. The specific roles of these authors are articulated in the ‘author contributions’ section.”

If your commercial affiliation did play a role in your study, please state and explain this role within your updated Funding Statement.

2. Please also provide an updated Competing Interests Statement declaring this commercial affiliation along with any other relevant declarations relating to employment, consultancy, patents, products in development, or marketed products, etc.

Within your Competing Interests Statement, please confirm that this commercial affiliation does not alter your adherence to all PLOS ONE policies on sharing data and materials by including the following statement: "This does not alter our adherence to  PLOS ONE policies on sharing data and materials.” (as detailed online in our guide for authors http://journals.plos.org/plosone/s/competing-interests) . If this adherence statement is not accurate and  there are restrictions on sharing of data and/or materials, please state these. Please note that we cannot proceed with consideration of your article until this information has been declared.

Please include both an updated Funding Statement and Competing Interests Statement in your cover letter. We will change the online submission form on your behalf.

Response: Thank you for your comments regarding the disclosure of commercial affiliations. The authors Fengwei An, Wei Liu, and Zhenglong Lv are employed by China Communications Construction Jiangsu Highway Engineering Co., Ltd.; Haoran Jiang and Wenpeng Du are employed by Shandong Pengcheng Road and Bridge Group Co., Ltd.; Yulong Zhao, Shujing Dong, Guohua Wu, and Hao Liang are employed by Shandong Jiaotong University; and Lei Fang is employed by Jining–Jizhou Expressway Co., Ltd.

These affiliations did not influence the study design, data collection and analysis, decision to publish, or preparation of the manuscript. The specific contributions of each author have been clearly described in the “Author Contributions” section. The Competing Interests statement in the manuscript has been revised accordingly.

Comment 9: Please ensure that you refer to Figure 1 in your text as, if accepted, production will need this reference to link the reader to the figure.

Response: Thank you for the reminder. We have ensured that Figure 2 is explicitly cited in the main text. Specifically, in Section 2.1.1 (Image Acquisition), we added the sentence: “The image acquisition setup for asphalt pavement surfaces is illustrated in Fig 2.” This revision ensures that all figures are properly cited in the text in chronological order.

Comment 10: .If the reviewer comments include a recommendation to cite specific previously published works, please review and evaluate these publications to determine whether they are relevant and should be cited. There is no requirement to cite these works unless the editor has indicated otherwise.

Response: Thank you for the reminder. We have carefully reviewed the publications suggested by the reviewers and evaluated their relevance to our study. Relevant references have been considered and cited where appropriate in the revised manuscript.

Comment 11:. Thank you for updating your data availability statement. You note that your data are available within the Supporting Information files, but no such files have been included with your submission. At this time we ask that you please upload your minimal data set as a Supporting Information file, or to a public repository such as Figshare or Dryad.Please also ensure that when you upload your file you include separate captions for your supplementary files at the end of your manuscript.As soon as you confirm the location of the data underlying your findings, we will be able to proceed with the review of your submission.

Response: Thank you for the guidance regarding the data availability statement. We have now archived the minimal data set supporting our findings in the Zenodo public repository.The materials are publicly accessible via the permanent DOI: 10.5281/zenodo.18870696. In accordance with your request, we have also added a 'Supporting Information' section at the end of the revised manuscript, providing clear captions and descriptions for these supplementary materials.

Comment 12:. It appears that your ORCiD iD has not been validated in your Editorial Manager account and we are unable to proceed until that step is complete.

Response: Thank you for the notification. We have now accessed the Editorial Manager account and successfully validated the ORCiD iD. We have confirmed that the green iD icon is now associated with the author profile as required. We apologize for the oversight and hope the submission can now proceed to the next stage.

Response to Comments from Reviewer #1

Your work on integrating U-Net segmentation with 3D reconstruction to assess pavement texture represents a meaningful advance toward automated, image-based skid resistance evaluation. The methodological approach is innovative, and the results demonstrate promising correlations with established measurement techniques.However, to meet the standards of reproducibility, rigor, and transparency expected by PLOS ONE, several important revisions are required. Below, I outline the key concerns that should be addressed in a revised submission.

Response:Thank you very much for your comments. We will do our best to improve the quality of the manuscript. Accordingly, the manuscript has been revised.

1. Methodological Transparency & Reproducibility

Comment 1: The 3D reconstruction pipeline is currently referenced as “Reference 27,” which is absent from the bibliography. Please provide a complete citation or a concise description of the SFM process, including software, parameters, and validation steps.

Response: Thank you for your valuable suggestion. To address your comment, we have supplemented the complete bibliographic information of Reference 30 and added a concise description of the SFM-based three-dimensional reconstruction procedure in the revised manuscript.Specifically, we now clarify the multi-view feature extraction and matching process, camera pose estimation, sparse and dense point cloud generation, mesh modeling, and texture mapping steps. In addition, the software platform (Agisoft Metashape), the SIFT-based feature matching method, and the validation procedures including scale calibration and model flatness testing have been explicitly stated.These additions enhance the methodological transparency and improve the reproducibility of the study.

References:

[30] DONG S J. Research on anti-skid performance detection and evaluation of asphalt pavement by integrating machine vision and deep learning [D]. Shandong Jiaotong University, 2025. https://doi.org/10.27864/d.cnki.gsjtd.2025.000140.

Comment 2: Specify the exact “local thresholding method” used for binarization (e.g., Otsu, adaptive mean) along with its parameters and justification.

Response: Thank you very much for the insightful suggestion. Accordingly, the manuscript has been revised.To address your comment, we have clarified the specific local thresholding method adopted in the reverse generation of reference binary images. The revised manuscript now explicitly states that a Gaussian-weighted adaptive local thresholding method was employed. In addition, we have supplemented a concise description of the implementation process, including extraction of the top-view image from the reconstructed three-dimensional pavement model, grayscale conversion using MATLAB, and adaptive threshold determination based on regional luminance distribution. The rationale for selecting this method has also been clarified, emphasizing its effectiveness in mitigating unev

Attachment Submitted filename: Response to Reviewers.docx

# Peer review: Decision Letter 1

**DOI:** 10.1371/journal.pone.0354127.r003

**Authors:** Baohua Guo

6 May 2026

PONE-D-25-68223R1

Binary Image Acquisition and Texture Parameter Calculation of Asphalt Pavement Based on a U-Net Model

PLOS One

Dear Dr. Zhao,

Thank you for submitting your manuscript to PLOS ONE. After careful consideration, we feel that it has merit but does not fully meet PLOS ONE’s publication criteria as it currently stands. Therefore, we invite you to submit a revised version of the manuscript that addresses the points raised during the review process.

Please submit your revised manuscript by Jun 20 2026 11:59PM. If you will need more time than this to complete your revisions, please reply to this message or contact the journal office at plosone@plos.org. When you're ready to submit your revision, log on to https://www.editorialmanager.com/pone/ and select the 'Submissions Needing Revision' folder to locate your manuscript file.

Please include the following items when submitting your revised manuscript:

- A letter that responds to each point raised by the academic editor and reviewer(s). You should upload this letter as a separate file labeled 'Response to Reviewers'.
- A marked-up copy of your manuscript that highlights changes made to the original version. You should upload this as a separate file labeled 'Revised Manuscript with Track Changes'.
- An unmarked version of your revised paper without tracked changes. You should upload this as a separate file labeled 'Manuscript'.

If you would like to make changes to your financial disclosure, please include your updated statement in your cover letter. Guidelines for resubmitting your figure files are available below the reviewer comments at the end of this letter.

If applicable, we recommend that you deposit your laboratory protocols in protocols.io to enhance the reproducibility of your results. Protocols.io assigns your protocol its own identifier (DOI) so that it can be cited independently in the future. For instructions see: https://journals.plos.org/plosone/s/submission-guidelines#loc-laboratory-protocols. Additionally, PLOS ONE offers an option for publishing peer-reviewed Lab Protocol articles, which describe protocols hosted on protocols.io. Read more information on sharing protocols at https://plos.org/protocols?utm_medium=editorial-email&utm_source=authorletters&utm_campaign=protocols.

As the corresponding author, your ORCID iD is verified in the submission system and will appear in the published article. PLOS supports the use of ORCID, and we encourage all coauthors to register for an ORCID iD and use it as well. Please encourage your coauthors to verify their ORCID iD within the submission system before final acceptance, as unverified ORCID iDs will not appear in the published article. *Only*  the individual author can complete the verification step; PLOS staff *cannot*  verify ORCID iDs on behalf of authors.

We look forward to receiving your revised manuscript.

Kind regards,

Baohua Guo

Academic Editor

PLOS One

Journal Requirements:

1. If the reviewer comments include a recommendation to cite specific previously published works, please review and evaluate these publications to determine whether they are relevant and should be cited. There is no requirement to cite these works unless the editor has indicated otherwise.

2. Please review your reference list to ensure that it is complete and correct. If you have cited papers that have been retracted, please include the rationale for doing so in the manuscript text, or remove these references and replace them with relevant current references. Any changes to the reference list should be mentioned in the rebuttal letter that accompanies your revised manuscript. If you need to cite a retracted article, indicate the article’s retracted status in the References list and also include a citation and full reference for the retraction notice.

[Note: HTML markup is below. Please do not edit.]

Reviewers' comments:

Reviewer's Responses to Questions

**Comments to the Author**

1. If the authors have adequately addressed your comments raised in a previous round of review and you feel that this manuscript is now acceptable for publication, you may indicate that here to bypass the “Comments to the Author” section, enter your conflict of interest statement in the “Confidential to Editor” section, and submit your "Accept" recommendation.

Reviewer #1: All comments have been addressed

Reviewer #3: All comments have been addressed

**********

2. Is the manuscript technically sound, and do the data support the conclusions?

The manuscript must describe a technically sound piece of scientific research with data that supports the conclusions. Experiments must have been conducted rigorously, with appropriate controls, replication, and sample sizes. The conclusions must be drawn appropriately based on the data presented.

Reviewer #1: Yes

Reviewer #3: Yes

**********

3. Has the statistical analysis been performed appropriately and rigorously?

Reviewer #1: Yes

Reviewer #3: Yes

**********

4. Have the authors made all data underlying the findings in their manuscript fully available?

The PLOS Data policy requires authors to make all data underlying the findings described in their manuscript fully available without restriction, with rare exception (please refer to the Data Availability Statement in the manuscript PDF file). The data should be provided as part of the manuscript or its supporting information, or deposited to a public repository. For example, in addition to summary statistics, the data points behind means, medians and variance measures should be available. If there are restrictions on publicly sharing data—e.g. participant privacy or use of data from a third party—those must be specified.

Reviewer #1: Yes

Reviewer #3: Yes

**********

5. Is the manuscript presented in an intelligible fashion and written in standard English?

PLOS ONE does not copyedit accepted manuscripts, so the language in submitted articles must be clear, correct, and unambiguous. Any typographical or grammatical errors should be corrected at revision, so please note any specific errors here.

Reviewer #1: Yes

Reviewer #3: Yes

**********

6. Review Comments to the Author

Please use the space provided to explain your answers to the questions above. You may also include additional comments for the author, including concerns about dual publication, research ethics, or publication ethics. (Please upload your review as an attachment if it exceeds 20,000 characters)

Reviewer #1: The revised manuscript has improved substantially compared with the earlier version. The authors have addressed many of the major concerns related to methodological transparency, reproducibility, data availability, figure referencing, novelty, limitations, and future deployment. The inclusion of the complete workflow, additional SFM reconstruction details, clarification of the U-Net training pipeline, and public repository information strengthens the manuscript.

The study presents a useful non-contact approach for asphalt pavement texture characterization by combining 3D reconstruction, inverse binary image generation, and U-Net-based semantic segmentation. The reported validation results, including an F1-score of 0.7214 and R² values of 0.85 and 0.86 for texture-related parameters, suggest that the proposed method has practical potential for pavement texture assessment.

However, a few issues should still be addressed before acceptance:

Statistical reporting should be strengthened.

The manuscript states that the correlations are statistically significant at the 95% confidence level, but the corresponding p-values, confidence intervals, or statistical test details are not clearly reported. Since the authors rely strongly on correlation-based validation, please provide p-values and/or 95% confidence intervals for the reported R²/correlation results. This would make the statistical interpretation more rigorous and transparent.

Agreement analysis remains limited.

The response indicates that Table 3 provides absolute and relative errors, but this is not fully equivalent to a formal agreement analysis. The authors may either include a Bland–Altman plot or briefly justify, in the manuscript itself, why the current error analysis is sufficient for evaluating agreement between the proposed image-based method and the sand patch method.

The claim related to skid resistance should be slightly qualified.

The manuscript validates the proposed method mainly against texture-depth-related measures rather than direct friction or skid resistance measurements. Therefore, statements such as “predict the skid resistance of asphalt pavements” should be revised to a more cautious form, such as “support texture-based assessment of pavement skid resistance” unless direct friction coefficient validation is included.

The binary cross-entropy equation should be carefully checked.

Please verify that the loss function is written in its standard form with correct brackets, negative sign, and all variables clearly defined. At present, the displayed equation appears to have a formatting issue around the logarithmic terms.

Model performance interpretation should be moderated.

The F1-score of 0.7214 indicates acceptable or promising segmentation performance, but the word “accurate” may be slightly strong without further IoU/Dice, confusion matrix, or class-wise performance reporting. Please consider adding IoU/Dice score or a short explanation of why this F1-score is adequate for the intended pavement texture application.

Dataset and validation scope should remain clearly limited.

The added discussion on illumination, pavement type, wet/contaminated surfaces, and lane-level scalability is useful. Please ensure that the conclusion does not overgeneralize beyond the available dataset, particularly because external validation under broader road and environmental conditions is still proposed as future work.

Minor language and formatting corrections are needed.

Please proofread the manuscript for spacing, punctuation, equation formatting, figure-caption consistency, and terminology. For example, maintain one consistent term for the reference method, preferably “sand patch method,” throughout the manuscript.

Overall, the manuscript is technically meaningful and has improved in response to the reviewers’ comments. I recommend acceptance after minor revision, provided the authors address the remaining statistical reporting, equation-formatting, and claim-qualification issues.

Reviewer #3: The presentation of the performance metrics (accuracy, precision, recall, and F1-score) should be improved for clarity and consistency. The current text contains minor inconsistencies and redundant phrasing, and it is unclear whether these metrics correspond to training, validation, or test data. A clearer, more structured reporting of these results is recommended, along with a more balanced interpretation of the model's performance.

In addition, the dataset size appears relatively small for training a deep learning model, and its potential impact on generalization should be discussed more explicitly. While the use of cross-validation helps mitigate this limitation, the description of the data partitioning strategy is unclear, as both k-fold cross-validation and a fixed training–validation split (8:2) are mentioned. The authors should clarify this aspect to ensure methodological transparency and reproducibility.

Finally, the statistical analysis could be slightly strengthened by including confidence intervals or p-values to support the reported correlations.

**********

7. PLOS authors have the option to publish the peer review history of their article (what does this mean?). If published, this will include your full peer review and any attached files.

If you choose “no”, your identity will remain anonymous but your review may still be made public.

**Do you want your identity to be public for this peer review?**  For information about this choice, including consent withdrawal, please see our Privacy Policy.

Reviewer #1: No

Reviewer #3: No

**********

[NOTE: If reviewer comments were submitted as an attachment file, they will be attached to this email and accessible via the submission site. Please log into your account, locate the manuscript record, and check for the action link "View Attachments". If this link does not appear, there are no attachment files.]

To ensure your figures meet our technical requirements, please review our figure guidelines: https://journals.plos.org/plosone/s/figures

You may also use PLOS’s free figure tool, NAAS, to help you prepare publication quality figures: https://journals.plos.org/plosone/s/figures#loc-tools-for-figure-preparation.

NAAS will assess whether your figures meet our technical requirements by comparing each figure against our figure specifications.

# Author comment: Author response to Decision Letter 2

**DOI:** 10.1371/journal.pone.0354127.r004

21 May 2026

Response to Reviewers

First of all, we would like to thank you for reviewing our paper and for the thoughtful comments, which help to improve the quality of our manuscript. Accordingly, we have revised our manuscript, and a lot of changes have been made to the manuscript. Also, the detailed responses to the comments are given below:

Response to Comments from Reviewer #1

Comment 1: The manuscript states that the correlations are statistically significant at the 95% confidence level, but the corresponding p-values, confidence intervals, or statistical test details are not clearly reported. Since the authors rely strongly on correlation-based validation, please provide p-values and/or 95% confidence intervals for the reported R²/correlation results. This would make the statistical interpretation more rigorous and transparent.

Response: Thank you very much for the insightful comment. We agree that providing detailed statistical information improves the rigor and transparency of the correlation analysis. Accordingly, the manuscript has been revised to include the statistical test details, corresponding p-values, and 95% confidence intervals for the reported correlation results.

Pearson correlation analysis was performed to evaluate the relationship between the extracted texture parameters and the measured MTD values. The results show that the correlation between fractal dimension and MTD-1 is statistically significant (r = 0.93, R² = 0.86, p < 0.001, 95% CI: 0.83-0.96), confirming the robustness and reliability of the proposed method.

These additions strengthen the statistical interpretation and improve the overall rigor of the manuscript.

Comment 2: The response indicates that Table 3 provides absolute and relative errors, but this is not fully equivalent to a formal agreement analysis. The authors may either include a Bland-Altman plot or briefly justify, in the manuscript itself, why the current error analysis is sufficient for evaluating agreement between the proposed image-based method and the sand patch method.

Response: Thank you very much for the valuable suggestion. We agree that agreement analysis is important for evaluating the consistency between the proposed image-based method and the conventional sand patch method.

Accordingly, a Bland-Altman analysis has been added in the revised manuscript to further evaluate the agreement between the two methods (Fig 13). The results show that most data points are distributed within the 95% limits of agreement, and the mean difference between the proposed method and the sand patch method remains relatively small, indicating good consistency between the two measurement approaches.

In addition, the absolute and relative error analyses presented in Table 3 further demonstrate that the differences between the proposed method and the standard sand patch method specified in the Quality Inspection and Assessment Standards for Highway Engineering. Volume 1. Civil Engineering (JTG F80/1) are relatively limited and remain within an acceptable range for pavement texture evaluation. These results suggest that the proposed image-based method can provide reliable support for pavement texture characterization under the investigated conditions.

References:

[1] JTG F80/1-2017, Quality Inspection and Assessment Standards for Highway Engineering. Volume 1. Civil Engineering.

Comment 3: The manuscript validates the proposed method mainly against texture-depth-related measures rather than direct friction or skid resistance measurements. Therefore, statements such as “predict the skid resistance of asphalt pavements” should be revised to a more cautious form, such as “support texture-based assessment of pavement skid resistance” unless direct friction coefficient validation is included.

Response: Thank you very much for your valuable suggestions. We agree that this study focuses primarily on parameters related to road surface texture, rather than direct measurements of friction coefficients or skid resistance. We have therefore revised the manuscript to use more cautious and accurate wording when describing the assessment of skid resistance.

Specifically, phrases such as ‘predicting skid resistance’ have been amended to ‘supporting skid resistance assessments based on road surface texture’ or ‘reflecting road surface texture characteristics associated with skid resistance’. Furthermore, the discussion section has been revised to explicitly state that the quantitative classification of road safety levels still requires further validation through friction-related indicators and relevant engineering standards.

Comment 4: Please verify that the loss function is written in its standard form with correct brackets, negative sign, and all variables clearly defined. At present, the displayed equation appears to have a formatting issue around the logarithmic terms.

Response: Thank you very much for the careful review. We agree that the original presentation of the binary cross-entropy loss function contained formatting inconsistencies, including unclear bracket arrangement and notation issues in the logarithmic terms.

Accordingly, the equation has been carefully checked and revised to the standard binary cross-entropy form. The negative sign, bracket structure, and normalization term have been corrected, and all variables involved in the equation are now explicitly defined in the manuscript to improve clarity and readability.

Comment 5: The F1-score of 0.7214 indicates acceptable or promising segmentation performance, but the word “accurate” may be slightly strong without further IoU/Dice, confusion matrix, or class-wise performance reporting. Please consider adding IoU/Dice score or a short explanation of why this F1-score is adequate for the intended pavement texture application.

Response: Thank you very much for your valuable suggestion. We agree that the interpretation of the segmentation performance should be presented in a more cautious and objective manner. Accordingly, in the revised manuscript, overly strong expressions such as “accurate” have been replaced with more appropriate descriptions, including “effective” or “acceptable.” In addition, following the reviewer’s suggestion, the Intersection over Union (IoU) metric has been added to Table 2, together with the corresponding calculation formula (Equation 6), in order to provide a more comprehensive evaluation of the spatial overlap performance of the model.

Although the obtained F1-score (0.7214) and IoU value (0.5642) indicate moderate pixel-level segmentation performance, this is mainly attributed to the inherent complexity of asphalt pavement textures, irregular void boundaries, and local shadow interference. Nevertheless, the achieved performance is considered sufficient for the intended purpose of this study. It should be emphasized that the primary objective of this work is to support pavement texture parameter extraction and texture-based pavement evaluation, rather than pursuing pixel-level semantic segmentation accuracy alone.

More importantly, although certain segmentation deviations still exist in locally complex regions, the Bland-Altman agreement analysis (Fig. 13) and the strong correlation with the sand patch method (R2=0.85 for MTD) indicate that these local pixel-level errors do not significantly affect the reliability of the final texture parameter calculations. Therefore, the current segmentation performance is considered sufficiently reliable to support the proposed pavement texture characterization and evaluation tasks.

Comment 6: The added discussion on illumination, pavement type, wet/contaminated surfaces, and lane-level scalability is useful. Please ensure that the conclusion does not overgeneralize beyond the available dataset, particularly because external validation under broader road and environmental conditions is still proposed as future work.

Response: Thank you very much for the valuable comment. We agree that the conclusions should remain consistent with the scope of the current dataset and experimental conditions.

Accordingly, the manuscript has been revised to avoid overgeneralization regarding the applicability and robustness of the proposed method. More cautious descriptions have been adopted throughout the discussion sections, emphasizing that the current findings were validated only under the investigated pavement and lighting conditions.

In addition, the revised manuscript now further clarifies that broader external validation under different pavement types, lighting conditions, wet or contaminated surfaces, and large-scale roadway environments is still required in future work before wider engineering application can be established.

Comment 7: Please proofread the manuscript for spacing, punctuation, equation formatting, figure-caption consistency, and terminology. For example, maintain one consistent term for the reference method, preferably “sand patch method,” throughout the manuscript.

Response: Thank you very much for the careful review and valuable suggestions. The manuscript has been thoroughly proofread and revised to improve language clarity and formatting consistency.

Specifically, spacing, punctuation, equation formatting, figure caption consistency, and terminology usage have been carefully checked and corrected throughout the manuscript. In addition, the terminology referring to the reference method has been standardized consistently as “sand patch method” across the entire paper.

Response to Comments from Reviewer #3

Comment 1: The presentation of the performance metrics (accuracy, precision, recall, and F1-score) should be improved for clarity and consistency. The current text contains minor inconsistencies and redundant phrasing, and it is unclear whether these metrics correspond to training, validation, or test data. A clearer, more structured reporting of these results is recommended, along with a more balanced interpretation of the model's performance.

Response: Thank you very much for the valuable suggestion. We agree that the presentation and interpretation of the performance metrics should be clearer and more objective.

Accordingly, the manuscript has been revised to explicitly clarify that the reported accuracy, precision, recall, and F1-score correspond to the test set results. The related descriptions have also been reorganized to improve clarity and consistency while avoiding redundant or overly subjective interpretations of model performance.

Comment 2: In addition, the dataset size appears relatively small for training a deep learning model, and its potential impact on generalization should be discussed more explicitly. While the use of cross-validation helps mitigate this limitation, the description of the data partitioning strategy is unclear, as both k-fold cross-validation and a fixed training–validation split (8:2) are mentioned. The authors should clarify this aspect to ensure methodological transparency and reproducibility.

Response: Thank you very much for your valuable comments. We agree that a clear description of the data partitioning strategy and a discussion on the dataset scale are essential for the transparency and reproducibility of deep learning research.

In the revised manuscript, we have clarified the data partitioning strategy to eliminate the ambiguity in the previous version. To ensure an objective evaluation of model performance while reducing the risk of overfitting and data leakage, a fixed hold-out validation strategy was adopted [1]. Specifically, a total of 180 asphalt pavement images were randomly divided into training and test sets at a ratio of 8:2 [2], where 144 images were used for model training and the remaining 36 images were reserved as an independent test set for performance evaluation. The test set was not involved during the training process. All images were resized to 512×512 pixels before training.

Regarding the dataset scale, although the number of pavement images is relatively limited compared with large-scale computer vision datasets, the collected images covered different pavement texture and lighting conditions, which helps improve dataset diversity under the investigated conditions. In addition, pavement texture segmentation mainly focuses on aggregate and void feature extraction rather than large-scale object recognition tasks. Previous studies have indicated that, for specialized engineering image segmentation tasks, relatively limited datasets can still provide effective feature representation and stable model performance.

More importantly, despite the relatively limited dataset size, the extracted texture parameters maintained strong correlations with the measured values obtained by the sand patch method, and the Bland–Altman analysis further demonstrated acceptable agreement between the proposed image-based method and the conventional method. These results indicate that the current dataset is sufficient to support pavement texture characterization under the investigated conditions. Nevertheless, broader external validation using larger and more diverse datasets is still required in future work to further evaluate the robustness and generalization capability of the proposed method. The above clarification has been added to the revised manuscript.

References:

[1] Hastie T, Tibshirani R, Friedman J. The Elements of Statistical Learning: Data Mining, Inference, and Prediction. 2nd ed. New York: Springer; 2009.

[2] He M, Lau TL. CrackHAM: A novel automatic crack detection network based on U-Net for asphalt pavement. IEEE Access. 2024;12:12655–12666.

Comment 3: Finally, the statistical analysis could be slightly strengthened by including confidence intervals or p-values to support the reported correlations.

Response: Thank you very much for the valuable suggestion. We agree that the inclusion of statistical significance indicators can further strengthen the reliability and transparency of the correlation analysis.

Accordingly, Pearson correlation significance analysis has been added in the revised manuscript. The corresponding p-values and 95% confidence intervals are now provided to support the statistical significance of the reported relationships between the extracted texture parameters and the measured MTD values. For example, the correlation analysis between the fractal dimension derived from binary images and the measured MTD values yielded r=0.93, R2=0.86, p<0.001, and a 95% confidence interval of 0.83-0.96, indicating a strong and statistically significant correlation.

Attachment Submitted filename: Response_to_Reviewers_auresp_2.docx

# Peer review: Decision Letter 2

**DOI:** 10.1371/journal.pone.0354127.r005

**Authors:** Baohua Guo

6 Jul 2026

<p>Binary Image Acquisition and Texture Parameter Calculation of Asphalt Pavement Based on a U-Net Model

PONE-D-25-68223R2

Dear Dr. Zhao,

We’re pleased to inform you that your manuscript has been judged scientifically suitable for publication and will be formally accepted for publication once it meets all outstanding technical requirements.

Within one week, you’ll receive an e-mail detailing the required amendments. When these have been addressed, you’ll receive a formal acceptance letter and your manuscript will be scheduled for publication.

An invoice will be generated when your article is formally accepted. Please note, if your institution has a publishing partnership with PLOS and your article meets the relevant criteria, all or part of your publication costs will be covered. Please make sure your user information is up-to-date by logging into Editorial Manager at Editorial Manager® and clicking the ‘Update My Information' link at the top of the page. For questions related to billing, please contact billing support.

If your institution or institutions have a press office, please notify them about your upcoming paper to help maximize its impact. If they’ll be preparing press materials, please inform our press team as soon as possible -- no later than 48 hours after receiving the formal acceptance. Your manuscript will remain under strict press embargo until 2 pm Eastern Time on the date of publication. For more information, please contact onepress@plos.org.

Kind regards,

Baohua Guo

Academic Editor

PLOS One

Additional Editor Comments (optional):

Reviewers' comments:

Reviewer's Responses to Questions

**Comments to the Author**

1. If the authors have adequately addressed your comments raised in a previous round of review and you feel that this manuscript is now acceptable for publication, you may indicate that here to bypass the “Comments to the Author” section, enter your conflict of interest statement in the “Confidential to Editor” section, and submit your "Accept" recommendation.

Reviewer #1: All comments have been addressed

Reviewer #4: All comments have been addressed

**********

2. Is the manuscript technically sound, and do the data support the conclusions?

The manuscript must describe a technically sound piece of scientific research with data that supports the conclusions. Experiments must have been conducted rigorously, with appropriate controls, replication, and sample sizes. The conclusions must be drawn appropriately based on the data presented.

Reviewer #1: Yes

Reviewer #4: Yes

**********

3. Has the statistical analysis been performed appropriately and rigorously?

Reviewer #1: Yes

Reviewer #4: Yes

**********

4. Have the authors made all data underlying the findings in their manuscript fully available?

The PLOS Data policy requires authors to make all data underlying the findings described in their manuscript fully available without restriction, with rare exception (please refer to the Data Availability Statement in the manuscript PDF file). The data should be provided as part of the manuscript or its supporting information, or deposited to a public repository. For example, in addition to summary statistics, the data points behind means, medians and variance measures should be available. If there are restrictions on publicly sharing data—e.g. participant privacy or use of data from a third party—those must be specified.

Reviewer #1: Yes

Reviewer #4: (No Response)

**********

5. Is the manuscript presented in an intelligible fashion and written in standard English?

PLOS ONE does not copyedit accepted manuscripts, so the language in submitted articles must be clear, correct, and unambiguous. Any typographical or grammatical errors should be corrected at revision, so please note any specific errors here.

Reviewer #1: Yes

Reviewer #4: (No Response)

**********

6. Review Comments to the Author

Please use the space provided to explain your answers to the questions above. You may also include additional comments for the author, including concerns about dual publication, research ethics, or publication ethics. (Please upload your review as an attachment if it exceeds 20,000 characters)

Reviewer #1: I have carefully reviewed the revised version of the manuscript titled “Binary Image Acquisition and Texture Parameter Calculation of Asphalt Pavement Based on a U-Net Model.” The authors have responded constructively and substantially to the previous review comments, and the revised manuscript shows clear improvement in methodological transparency, statistical reporting, and scientific interpretation.

The inclusion of p-values and 95% confidence intervals strengthens the statistical validation of the reported correlations. The addition of Bland-Altman analysis is also valuable, as it provides a more appropriate assessment of agreement between the proposed image-based texture measurement method and the conventional sand patch method. The authors have also improved the reporting of segmentation performance by adding the IoU metric and by interpreting the F1-score more cautiously and objectively.

The clarification of the dataset partitioning strategy, including the 8:2 training-test split and the limited dataset size, improves reproducibility. I also appreciate that the authors have revised the language related to skid resistance, avoiding overstatement and more appropriately framing the work as supporting texture-based skid-resistance assessment rather than directly predicting friction performance. The limitation regarding broader external validation under varied pavement, illumination, wet, contaminated, and large-scale roadway conditions has also been adequately acknowledged.

Overall, the revised manuscript has addressed the major scientific and presentation concerns raised during review. The study is methodologically sound within its stated scope and offers a useful image-based approach for pavement texture characterization. Only routine editorial proofreading may be needed to ensure final consistency in language, formatting, and figure/table presentation.

Recommendation: Accept.

Reviewer #4: (No Response)

**********

7. PLOS authors have the option to publish the peer review history of their article (what does this mean?). If published, this will include your full peer review and any attached files.

If you choose “no”, your identity will remain anonymous but your review may still be made public.

**Do you want your identity to be public for this peer review?**  For information about this choice, including consent withdrawal, please see our Privacy Policy.

Reviewer #1: No

Reviewer #4: No

**********

# Editor report: Acceptance letter

**DOI:** 10.1371/journal.pone.0354127.r006

**Authors:** Baohua Guo

PONE-D-25-68223R2

PLOS One

Dear Dr. Zhao,

I'm pleased to inform you that your manuscript has been deemed suitable for publication in PLOS One. Congratulations! Your manuscript is now being handed over to our production team.

At this stage, our production department will prepare your paper for publication. This includes ensuring the following:

* All references, tables, and figures are properly cited

* All relevant supporting information is included in the manuscript submission,

* There are no issues that prevent the paper from being properly typeset

You will receive further instructions from the production team, including instructions on how to review your proof when it is ready. Please keep in mind that we are working through a large volume of accepted articles, so please give us a few days to review your paper and let you know the next and final steps.

Lastly, if your institution or institutions have a press office, please let them know about your upcoming paper now to help maximize its impact. If they'll be preparing press materials, please inform our press team within the next 48 hours. Your manuscript will remain under strict press embargo until 2 pm Eastern Time on the date of publication. For more information, please contact onepress@plos.org.

You will receive an invoice from PLOS for your publication fee after your manuscript has reached the completed accept phase. If you receive an email requesting payment before acceptance or for any other service, this may be a phishing scheme. Learn how to identify phishing emails and protect your accounts at https://explore.plos.org/phishing.

If we can help with anything else, please email us at customercare@plos.org.

Thank you for submitting your work to PLOS ONE and supporting open access.

Kind regards,

PLOS ONE Editorial Office Staff

on behalf of

Dr. Baohua Guo

Academic Editor

PLOS One
